# Developmental Regulation of Diacylglycerol Acyltransferase Family Gene Expression in Tung Tree Tissues

**DOI:** 10.1371/journal.pone.0076946

**Published:** 2013-10-11

**Authors:** Heping Cao, Jay M. Shockey, K. Thomas Klasson, Dorselyn C. Chapital, Catherine B. Mason, Brian E. Scheffler

**Affiliations:** 1 U.S. Department of Agriculture, Agricultural Research Service, Southern Regional Research Center, Commodity Utilization Research Unit, New Orleans, Louisiana, United States of America; 2 U.S. Department of Agriculture, Agricultural Research Service, Genomics and Bioinformatics Research Unit, Stoneville, Mississippi, United States of America; University Paris South, France

## Abstract

Diacylglycerol acyltransferases (DGAT) catalyze the final and rate-limiting step of triacylglycerol (TAG) biosynthesis in eukaryotic organisms. DGAT genes have been identified in numerous organisms. Multiple isoforms of DGAT are present in eukaryotes. We previously cloned DGAT1 and DGAT2 genes of tung tree (*Vernicia fordii*), whose novel seed TAGs are useful in a wide range of industrial applications. The objective of this study was to understand the developmental regulation of DGAT family gene expression in tung tree. To this end, we first cloned a tung tree gene encoding DGAT3, a putatively soluble form of DGAT that possesses 11 completely conserved amino acid residues shared among 27 DGAT3s from 19 plant species. Unlike DGAT1 and DGAT2 subfamilies, DGAT3 is absent from animals. We then used TaqMan and SYBR Green quantitative real-time PCR, along with northern and western blotting, to study the expression patterns of the three DGAT genes in tung tree tissues. Expression results demonstrate that 1) all three isoforms of DGAT genes are expressed in developing seeds, leaves and flowers; 2) DGAT2 is the major DGAT mRNA in tung seeds, whose expression profile is well-coordinated with the oil profile in developing tung seeds; and 3) DGAT3 is the major form of DGAT mRNA in tung leaves, flowers and immature seeds prior to active tung oil biosynthesis. These results suggest that DGAT2 is probably the major TAG biosynthetic isoform in tung seeds and that DGAT3 gene likely plays a significant role in TAG metabolism in other tissues. Therefore, DGAT2 should be a primary target for tung oil engineering in transgenic organisms.

## Introduction

Triacylglycerols (TAGs) are the primary form of energy storage in eukaryotes. They also serve as a reservoir of fatty acids for membrane biogenesis of the cells and lead to obesity when excessively accumulated in adipose tissues [1]. Understanding plant TAG biosynthesis will help to create new oilseed crops with value-added properties [2]. Our project focuses on engineering oilseed crops to produce industrially useful oils. One such oil is the drying oil found in the seeds of tung tree (*Vernicia fordii).*

Tung tree is a tropical plant with a very limited growing area in the southeastern United States [3], [4]. Most of the tung orchards were destroyed by hurricanes, including Hurricanes Camille in 1969, and Katrina and Rita in 2005. Tung tree produces large seeds containing about 50–60% oil (dry weight basis) with approximately 80 mole % α-eleostearic acid (9*cis*, 11*trans*, 13*trans* octadecatrienoic acid) [5]. Due to the three unique conjugated double bonds in eleostearic acid, tung oil is readily oxidized. Dried tung oil is impervious to heat, moisture, dust and many chemical challenges, and unlike other drying oils, does not darken with age. These properties underline the value of tung oil as a drying ingredient in paints, varnishes, and other coatings and finishes [6], [7]. Tung oil has also been used as a raw material to produce biodiesel [8]–[12], polyurethane and wood flour composites [13], thermosetting polymer [14] and repairing agent for self-healing epoxy coatings [15]. The losses of tung orchards due to hurricanes have spurred interest in trying to preserve a reliable domestic source of tung oil in the U.S. by transferring the genetic components of the tung oil biosynthetic pathway into traditional, temperate oilseeds.

Several genes involved in tung oil biosynthesis have been identified in tung trees. These genes code for diacylglycerol acyltransferase 1 and 2 (DGAT1 and DGAT2) [16], delta-12 oleic acid desaturase (FAD2) [17], delta-12 fatty acid conjugase (FADX) [17], omega-3 fatty acid desaturase (FAD3) [18], acyl-CoA binding proteins (ACBPs) [19], cytochrome b5 isoforms (Cb5s) [20], cytochrome b5 reductase [21], glycerol-3-phosphate acyltransferases (GPATs) [22], aquaporin [23], glutaredoxin [23] and oleosins [24].

DGATs catalyze the final and rate-limiting step of TAG biosynthesis in eukaryotic organisms [25], [26]. DGATs esterify *sn*-1,2-diacylglycerol with a long-chain fatty acyl-CoA. At least two forms of DGATs are present in plants and animals, including DGAT1 and DGAT2 isoforms of tung tree [16], [27]. Recently, diverged forms of DGATs are reported, such as DAcT in burning bush (*Euonymus alatus*) [28], soluble DGAT in peanut [29] and a bifunctional DGAT in *Acinetobacter calcoaceticus* ADP1 [30]. Topology analysis, hydropathy plots and sequence analysis indicate that DGATs are integral membrane proteins [16], [31], [32] with more than 40% of the total hydrophobic amino acid residues [33]. The DGAT1s and DGAT2s have similar properties and amino acid composition except that DGAT1s are approximately 20 kDa larger than DGAT2s [33]. DGAT isoforms have non-redundant functions in TAG biosynthesis in species such as mice [34] and tung tree [16], [24].

There are many knowledge gaps in tung oil biosynthesis. Does additional form of DGAT exist in tung tree? How many isoforms of DGATs are expressed in tung seeds? What are the relative expression levels of these isoforms in tung seeds? How do DGAT expression levels correlate with tung oil accumulation in the seeds? Some of these questions could be answered by quantitative real-time-PCR (qPCR), a widely used method for gene expression analysis due to its large dynamic range, tremendous sensitivity, high sequence-specificity, little to no post-amplification processing and sample high-throughput [35]–[39]. TaqMan and SYBR Green qPCR are two frequently used qPCR techniques. TaqMan utilizes a fluorogenic single-stranded oligonucleotide probe that binds to specific DNA sequence between the two sequence-specific PCR primers, resulting in specific PCR products [40]. SYBR Green dye intercalates into double-stranded DNA to monitor the amplification of target gene specifically initiated by DNA-specific primers [41]. Both TaqMan and SYBR Green qPCR are reliable assays for quantitative gene expression in tung tree tissues [42] and animal cells [43].

The objective of this study was to understand the developmental regulation of DGAT family gene expression in tung tree. We cloned tung DGAT3 and used TaqMan and SYBR Green qPCR assays to evaluate the relative abundance and tissue distribution of the three DGAT mRNAs in seeds, leaves and flowers of tung tree. Northern blotting was used to confirm the expression pattern of DGATs in tung tissues. We raised antibodies against recombinant DGAT2 protein and used them to detect endogenous DGAT2 protein in developing tung seeds. Finally, we analyzed oil accumulation, whose profile was well correlated with DGAT2 mRNA and protein levels in developing tung seeds.

## Materials and Methods

### Plant Materials

Tung trees were grown in the American Tung Oil Corporation orchard in Lumberton, Mississippi. Tung fruits were collected every week beginning June 23, 2006, approximately 9 weeks after full bloom and 1 month before the initiation of storage oil synthesis. Company officer John Corley granted permission of this field study. Tung tree seeds were removed and kernels were immediately frozen in liquid N_2_ and stored at –80°C.

### Cloning of the Tung DGAT3 Gene

The 3′ portion of the tung DGAT3 gene was identified from double-stranded cDNA samples prepared from mid-development (time point 6) tung seeds, as described previously [19]. The remaining sequence at the 5′ end of the open-reading frame was determined by two rounds of adaptor anchored PCR from tung genomic DNA, as described by the manufacturer (GenomeWalker kit, Clontech), using the provided adaptor primers and the gene-specific primers VfDGAT3-5GW1 (5′-GACCCTGACATTAGGACCGTCCCTG-3′) and VfDGAT3-5GW2 (5′-CCCTGCACTTGCCCATACACTTGCA-3′). The likely initiator ATG codon was chosen from the genomic sequence using the NetStart server (http://www.cbs.dtu.dk/services/NetStart/) [44] and by alignment of the possible predicted amino acid translations to other known or predicted plant DGAT3 protein sequences available in public databases. The full-length ORF was amplified by RT-PCR from the same samples used to generate the 454 data using the primers VfDGAT3-ATG (5′-ATGGAGCTTTCCGGCGTGGCTCTC-3′) and VfDGAT3-stop (5′-CTAAGATGCAGGGGCCAGACCCAAATG-3′). The sequence of tung DGAT3 was submitted to Genbank under the accession number KC993814.

### Computational Methods

DGAT3 sequences from other organisms were obtained from BlastP searches [45], [46] using tung tree DGAT3 sequence against the National Center for Biotechnology Information (NCBI)’s non-redundant protein sequence databases (http://blast.ncbi.nlm.nih.gov/Blast.cgi). A total of 31 full-length and near full-length DGAT3s were obtained from 23 organisms including plants (such as *Arabidopsis*, barley, castor bean, corn, rape, rice, sorghum, soybean, tomato and tung tree) and fungi. The properties and amino acid compositions of DGAT3s were analyzed using Vector NTI software (Life Technologies, Carlsbad, CA) [47]. Statistics were performed using Microsoft Excel. Phylogenetic analysis was used to study the presumed evolutionary relationships among the DGAT3 proteins from the selected organisms. This analysis was performed using the Vector NTI software based on the neighbor-joining method of Saitou and Nei [48]. Multiple sequence alignment was performed using the ClustalW algorithm [49], [50] of the AlignX program of the Vector NTI software. This method is based on algorithms that assign scores to aligned residues and detect sequence similarities. Identical amino acid residues in alignment have higher scores than those not identical and less similar residues.

### RNA Isolation

Tung seeds were ground into powder with mortar and pestle under liquid nitrogen. Total RNAs were isolated by Spectrum Plant Total RNA Kit (Sigma, St. Louis, MO) as described [42]. Total RNAs from tung seeds, leaves and flowers were also extracted using the hot borate method [51]. RNA concentrations and integrity were determined using RNA 6000 Nano Assay Kit and the Bioanalyzer 2100 (Agilent Technologies, Santa Clara, CA) with RNA 6000 Ladder as the standards [52]. The RNA isolated were shown to be high quality as determined by high rRNA ratio (28S/18S = 1.9) and the RNA integrity number (RIN = 8.7) (data not shown and [42]).

### Quantitative Real-time PCR Analysis

TaqMan and SYBR Green qPCR assays followed the MIQE guidelines: minimum information for publication of quantitative real-time PCR experiments [53]. PCR primers and TaqMan probes were designed using Primer Express software (Applied Biosystems, Foster City, CA). The *T*_m_s of the primers were variable to a few degrees but the *T*_m_s for the probes were generally 10 degrees higher than the corresponding primers. They were synthesized by Biosearch Technologies, Inc. (Navato, CA). The amplicon sizes and the nucleotide sequences (5′ to 3′) of the forward primers, TaqMan probes (TET – BHQ1) and reverse primers, respectively, are described in Table 1.

**Table 1:** DGAT and reference gene expression analyzed by qPCR and the nucleotide sequences of qPCR primers and TaqMan probes.

No	mRNA	Name	Accessionnumber	Amplicon(bp)	Forward primer(5′ to 3′)	TaqMan probe(5′ to 3′)	Reverse primer(5′ to 3′)
1	DGAT1	Diacylglycerol acyltransferase 1	DQ356680	70	TGGTTGCTCCCACATTGTGT	ACCAGCCAAGTTATCCTCGAACTGCATCC	ACCACCCAACCCTTTCGAA
12	DGAT2	Diacylglycerol acyltransferase 2	DQ356682	80	TGCATGTCGTGGTGGGTAGA	AAGCAAAATCCACAGCCTACCGCTGAA	TCTCTGTACTTCCGCAACCT
13	DGAT3	Diacylglycerol acyltransferase 3	Unpublished	64	AAGTGCAGGGACGGTCCTAA	TCAGGGTCAATGCTG	TGGGTGGAGTTGTGATTGGA
4	Gapdh	glyceraldehyde 3 phosphate dehydrogenase	Unpublished	66	TGGAAGCACTCCGATGTGAA	TCAAGGATGAGAAGACC	AGTAACTGGCTTCTCACCAAAGAGA
5	Rpl19b	Ribosomal protein 19b	FJ362591	70	GCGGAGAATGCGTGTTCTG	CCTGCTGCGCAAATACCGGGAA	CATGTGCTTGTCAATTTTTTTGG
6	Ubl	ubiquitin protein ligase	Unpublished	61	AGCTGGACCCAGAAGTATGCA	TGGGTTAACAGTGCTGC	AGCCCTCCCTATCACACACAA

The cDNAs were synthesized from total RNAs using SuperScript II reverse transcriptase (Life Technologies). The cDNA synthesis mixture (20 µl) contained 5 µg total RNA, 2.4 µg oligo(dT)_12–18_ primer, 0.1 µg random primers, 500 µM dNTPs, 10 mM DTT, 40 units RNaseOUT and 200 units SuperScript II reverse transcriptase in 1X first-strand synthesis buffer. The cDNA synthesis reactions were incubated at 42°C for 50 min. The cDNA samples were stored in −80°C freezer and diluted with water to 1 ng/µl before qPCR analyses.

TaqMan qPCR reaction mixtures contained variable amounts of total RNA-derived cDNAs (2.5, 5, 12.5 and 25 ng), 200 nM each of the forward primer, reverse primer and TaqMan probe, and 1× Absolute QPCR Mix (ABgene House, Epson, Surrey, UK) [52]. The thermal cycling conditions for TaqMan qPCR were as follows: 2 min at 50°C and 15 min at 95°C, followed by 40–50 cycles at 95°C for 15 s and 60°C for 60 s. SYBR Green qPCR reaction mixtures contained variable amounts of total RNA-derived cDNAs, forward primer, reverse primer and 1X iQ SYBR Green Supermix (Bio-Rad Laboratories, Hercules, CA). The thermal cycle conditions for SYBR Green qPCR were as follows: 3 min at 95°C, followed by 40 cycles at 95°C for 10 s, 65°C for 30 s and 72°C for 30 s. The qPCR reactions were performed in 96-well clear plates sealed by adhesives with a CFX96 real-time system-C1000 Thermal Cycler (Bio-Rad Laboratories). The ΔΔ*C_T_* method of relative quantification was used to determine the fold change in expression [54]. This was done by first normalizing the resulting threshold cycle (*C_T_*) values of the target mRNAs to the *C_T_* values of the internal reference controls including tung 60 s ribosome protein L19 (Rpl19b), glyceraldehyde 3 phosphate dehydrogenase (Gapdh) and ubiquitin protein ligase (Ubl) in the same samples (Δ*C_T_* = *C_T_*_Target_ - *C_T_*_ref_). Recent studies showed that Rpl19b was the most stably expressed gene, followed closely by Ubl, and Gapdh was the worst among the three genes under optimized qPCR assay conditions [55], [56]. The Δ*C_T_* of the target mRNA was further normalized with the calibrator (ΔΔ*C_T_* = Δ*C_T_*_Target_ - Δ*C_T_*_cal_). The fold change in expression was then obtained (2^−ΔΔ*CT*^). The amplification efficiency of qPCR assay is estimated on the basis of the equation *E* = (10^−1/slope^ − 1)×100 [57].

### Southern Blotting

The gene copy number of DGAT3 along with DGAT1 and DGAT2 in the tung genome was determined by Southern blotting. Five micrograms of tung genomic DNA were digested with either HindIII, NcoI, or SacI. The genomic DNA fragments were separated by agarose gel, transferred onto membranes and detected with PCR-generated, digoxygenin-labeled probes using PCR DIG probe synthesis kit (Roche Diagnostics, Indianapolis, IN), as described in Shockey et al., 2006 [16].

### Northern Blotting

Total RNA was extracted from tung leaves, flowers and developing seeds using the hot borate method [51]. Seven and one-half micrograms of total RNA were separated on MOPS–formaldehyde agarose gels, transferred to membranes and probed with digoxigenin-labeled probes covering the 3′ 1181 bases of the ORF for DGAT1 and the full-length ORFs for DGAT2 and DGAT3, as described previously [16]. A LAS-3000Plus digital imaging system (Fuji Medical Systems, Stamford, CT) was used for signal visualization and digital data capture.

### DGAT2 Antibody Production and Purification

The antigen rDGAT2 was purified from overexpressed *E. coli* as reported [58] and concentrated with a Centricon-10 concentrator (Amicon, Beverly, MA) to a protein concentration of 1 mg/ml. Anti-rDGAT2 serum was produced in rabbits immunized with the purified rDGAT2 fusion protein using a similar procedure as described previously [59] (Covance Research Products, Denver, PA). Briefly, 250 µg of the purified antigen was diluted into 0.5 ml in PBS, mixed with 0.5 ml of Freund’s complete adjuvant and injected into a female New Zealand white rabbit. Five boosts of 125 µg each of the purified antigen in Freund’s incomplete adjuvant were performed every 4 weeks following the primary injection. The crude antiserum was affinity-purified against rDGAT2 using a procedure similar to that for purifying anti-GST-hTTP antibodies [60]. Briefly, *E. coli* strain BL21(DE3) was transformed with expression plasmid pMBP-DGAT2. rDGAT2 expression was induced by IPTG. The cells were homogenized by sonication. Following centrifugation at 10,000 *g* for 10 min, proteins in the pellet were separated by preparative SDS–PAGE and transferred onto nitrocellulose membranes. Membrane strips corresponding to rDGAT2 were excised and used to affinity purify anti-rDGAT2 from the crude antiserum following the procedures as described [61]. Antibody production in animals was carried out in strict accordance with the recommendations in the Guide for the Care and Use of Laboratory Animals of the National Institutes of Health. The protocol was approved by the Committee on the Ethics of Animal Experiments of Covance Research Products Inc. All surgery was performed subcutaneously and animals were sacrificed under sodium pentobarbital anesthesia, and all efforts were made to minimize suffering.

### Microsomal Membrane Preparation

Microsomal membranes were isolated from tung seeds by differential centrifugation [62]. Briefly, tung seeds were ground with a mortar and pestle in homogenization buffer containing 0.4 M sucrose, 50 mM 3-(N-morpholino) propanesulfonic acid (MOPS), pH 6.9, 10 mM DTT, 1 mM EDTA, 0.1 mM PMSF and 0.1% *(w/v)* bovine serum albumin (BSA). The seed homogenates were centrifuged sequentially at 2,000 *g* for 10 min, 10,000 *g* for 20 min and 100,000 *g* for 60 min. The membrane pellet at 100,000 *g* was microsomal membranes. The microsomal membranes and the 10,000 *g* pellet were suspended in a buffer containing 10 mM Tricine, pH 7.2, 1 mM EDTA and 0.2 M sucrose (TES buffer).

### Protein Determination, SDS–PAGE and Immunoblotting

Protein concentrations were determined with the Bradford method using the Protein Assay Dye Reagent Concentrate (Bio-Rad Laboratories) following 0.5 M NaOH treatment of the protein samples [62]. Proteins were separated by SDS–PAGE (4–20%) (Life Technologies) and transferred onto nitrocellulose membranes for immunoblotting followed previously described procedures [63] using SuperSignal West Pico Chemiluminescent Substrate (Pierce, Rockford, IL). The primary antibodies used were affinity-purified anti-rDGAT2 as described above. The secondary antibodies used were affinity-purified goat anti-rabbit IgG (H+L) horseradish peroxidase conjugate with human IgG absorbed (Bio-Rad Laboratories), using 1∶5000 or 1∶10,000 dilutions in TTBS.

### Lipid Extraction and Analysis

Prior to analysis, seeds were cracked, hulls were removed and the remaining seeds were coarsely chopped and freeze-dried. The freeze-dried samples were further chopped to less than 0.84 mm and stored at –20°C until final analysis. Total seed oil content was determined by weight using a Soxhlet extractor (Tecator Soxtec System HT 1043, Foss, Eden Prairie, MN) with 3 g of seed and 40 ml of petroleum ether. Seed lipid composition was determined after converting all lipids to methyl esters by combining five chrome stain steel balls (2.3 mm, BioSpec Products, Bartlesville, OK) with 50 mg of seed and 500 µl of sodium methoxide (0.5 M in methanol, Sigma-Aldrich, St. Louis, MO) in a 2-ml round-bottom centrifuge tube. The tubes were then pulverized twice for 5 min each, with a 10 min cooling period in between (Disruptor Genie, Scientific Instruments, Bohemia, NY). Following a final 10-min waiting period, the content was filtered through Costar Spin-X 0.45-µm-pore-size nylon filter (Corning, Tewksbury, MA). The filtrate was combined with 500 µl saturated NaCl solution and 1250 µl heptane and vortexed for 10 sec. After a 10-min wait, the organic phase was transferred into a vial for gas chromatography automatic sampling. The samples were injected into a gas chromatograph (Model 6890, Agilent, Santa Clara, CA) equipped with a 30 m long capillary column (Supelco SP-2380, 0.20 µm film thickness, Bellefonte, PA) and a flame ionization detector. The injector and detector temperatures were held constant at 220°C and the oven was increased from 160°C to 200°C at 4°C/min and then held at 200°C for 10 min. The peaks corresponding to lipid fatty acids were identified by comparing to residence times for fatty acid methyl ester standards (Supleco).

## Results

### Cloning of DGAT3 Gene in Tung Tree

Anonymous tung seed cDNA sequences from week 6 were generated and analyzed by pyrosequencing (“454”) technology as described previously [19]. Comparisons of this dataset to existing databases using BLAST revealed a partial sequence highly similar to the sequence coding for the soluble isoform of DGAT from peanut (AhDGAT3 [29]). From this partial sequence, gene-specific primers were designed and used to isolate and confirm the DNA sequence of the remaining portion of the 3′ end of the full-length cDNA by rapid amplification of cDNA ends (3′ RACE). The remaining 5′ portion of the gene was amplified by genome walking as described previously [16].

**Figure 1:**
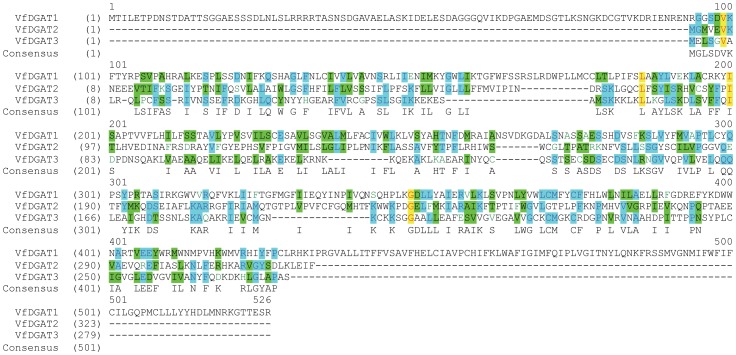
Tung tree DGAT amino acid sequence alignment. The full-length protein sequences of tung DGAT1, DGAT2 and DGAT3 were aligned using the ClustalW algorithm of the AlignX program of the Vector NTI software. The nucleotide sequence alignment of tung tree DGATs is shown in Figure S1.

This tung DGAT (named DGAT3) shared some conservation with either tung DGAT1 or DGAT2 at the protein level; only four amino acid residues were completely conserved amongst the three tung DGAT enzymes (Figure 1), although more nucleotide sequence conservations were present among the three DGAT genes (Figure S1). A striking feature of tung DGAT3 was its abnormally short length of only 278 amino acid residues. DGAT3 protein was the smallest among the three tung DGAT isoforms, with a calculated molecular mass of 19 kDa and 6 kDa less than DGAT1 and DGAT2, respectively (Table 2). Tung DGAT3 was also the smallest among the plant DGAT3s and was 82 residues shorter than the mean of 27 DGAT3 proteins from 19 plant species (Table 2). Another significant difference between DGAT3 and DGAT1 or DGAT2 was the percentage of hydrophobic residues. DGAT3 had approximately 31% of hydrophobic residues whereas DGAT1 had 41% and DGAT2 had 43% (Table 2).

**Table 2:** The properties and amino acid composition of the tung DGAT family and DGAT3 subfamily with 27 DGATs from 19 plant species.

Properties and amino acidcomposition (% by frequency)	DGAT1 (tung tree)	DGAT2 (tung tree)	DGAT3 (tung tree)	DGAT3 (average from 19 plant species)
Length (amino acid residue)	526	322	278	360±73
Molecular weight (Da)	59773	36726	30376	38705±7622
Isoelectric point (PI)	8.91	9.24	8.74	7.76±1.47
Charge at pH 7	11.78	8.44	7.12	3.96±7.43
Charged (RKHYCDE) (%)	27.00	23.60	36.25	38.57±1.73
Acidic (DE) (%)	7.98	7.14	12.23	14.85±1.74
Basic (KR) (%)	10.08	9.63	14.75	17.66±1.54
Polar (NCQSTY) (%)	25.86	21.74	27.34	25.23±2.62
Hydrophobic (AILFWV) (%)	41.06	43.48	31.65	28.09±1.88

Gene copy number in the tung genome was assessed by genomic Southern blotting, with detection by PCR-generated, digoxygenin-labeled probes. Tung genomic DNA was digested with either HindIII, NcoI, or SacI. The open reading frame for tung DGAT3, contains one site for NcoI (bp 109) and SacI (bp 318). the NcoI lane reveals a single hybridizing fragment, at approximately 4.5 kb (data not shown). While at least two hybridizing fragments would have been expected, given the internal NcoI site in tung DGAT3, no additional hybridizing fragments were observed (data not shown). These results suggest that the DGAT3, like DGAT1 and DGAT2 [16], exists as a single copy gene in the tung genome.

### Tung DGAT3 Belongs to the Soluble DGAT Subfamily in Plants

**Figure 2:**
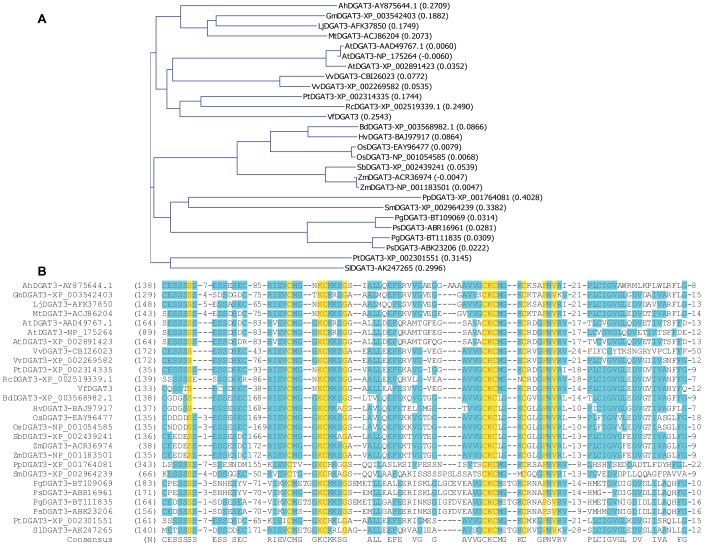
Phylogenetic analysis and identification of amino acid residues and sequence motifs conserved in DGAT3s. (A) Phylogenetic analysis. The presumed evolutionary relationships among the 27 DGATs from 19 organisms were analyzed by phylogenetic analysis. The numbers in the parenthesis following DGAT names are the calculated distance values, which reflect the degree of divergence between all pairs of DGAT sequences analyzed. (B) Identification of amino acid residues and sequence motifs conserved in DGAT3s. Multiple sequence alignment was performed using the ClustalW algorithm of the AlignX program of the Vector NTI software. Each DGAT sequence name is on the left of the alignment followed by the position of amino acid residue of DGAT protein sequence in the alignment. The numbers before, in the middle and after the amino acid residues in the sequence alignment represent the number of residues in the divergent region as previously used [63]. The letters at the bottom of the alignment are the consensus residues. Color codes for amino acid residues are as follows: 1) red on yellow: consensus residue derived from a completely conserved residue at a given position; 2) blue on cyan: consensus residue derived from the occurrence of greater than 50% of a single residue at a given position; 3) black on white: non-similar residues. The complete sequence alignment is shown in Figure S2. The abbreviations of the organisms are: Ah, *Arachis hypogaea* (peanut); At, *Arabidopsis thaliana*; Bd, *Brachypodium distachyon;* Gm, *Glycine max* (soybean); Hv, *Hordeum vulgare (barley)*; Lj, *Lotus japonicas*; Mt, *Medicago truncatula*; Os, *Oryza sativa* (rice); Pg, *Picea glauca* (white spruce); Pp, *Physcomitrella patens*; Ps, *Picea sitchensis* (sitka spruce); Pt, *Populus trichocarpa*; Rc, *Ricinus communis* (caster bean); Sb, *Sorghum bicolor* (sorghum); Sl, *Solanum lycopersicum* (tomato); Sm, *Selaginella moellendorffii*; Vf, *Vernicia fordii* (tung tree); Vv, *Vitis vinifera* (grape); Zm, *Zea mays* (corn).

Database search using tung DGAT3 amino acid sequence identified 26 DGAT3s from 18 other plant species including the well-studied soluble DGAT3 from peanut [29]. Phylogenic analysis showed that tung DGAT3 was most closely related to castor bean DGAT3 (Figure 2A). Multiple sequence alignment was further performed to identify conserved amino acid residues and sequence motifs in the DGAT3 subfamily to strengthen our conclusion that the cloned tung DGAT3 is full-length and belongs to the soluble DGAT subfamily. Among the 27 DGAT3s from 19 plant species, 11 amino acid residues were completely conserved, which corresponded to 3.0% of the total 360 residues of DGAT3s (Figure 2B). This percentage of completely conserved residues in DGAT3s was much smaller than DGAT1s (8.0%) or DGAT2s (4.7%) [33]. Ten of the 11 completely conserved residues were located within the last 100 residues from the carboxyl termini of DGAT3 proteins and only one at the amino termini of these proteins (Figure 2B). This suggests that the catalytic domains of DGAT3s are probably located at the carboxyl termini, similar to those of DGAT1s and DGAT2s [33]. Tung DGAT3 protein contained all the 11 completely conserved residues and was highly similar to DGAT3s from other species (Figure 2B). In addition, the amino and carboxyl termini of tung DGAT3 were properly aligned with those of the other plant DGAT3s, which were highly conserved among the DGAT3s (Figure S2). These sequence analyses clearly support our conclusion that the cloned DGAT3 from tung tree is full-length.

### TaqMan qPCR Optimization for DGAT Gene Expression

**Figure 3:**
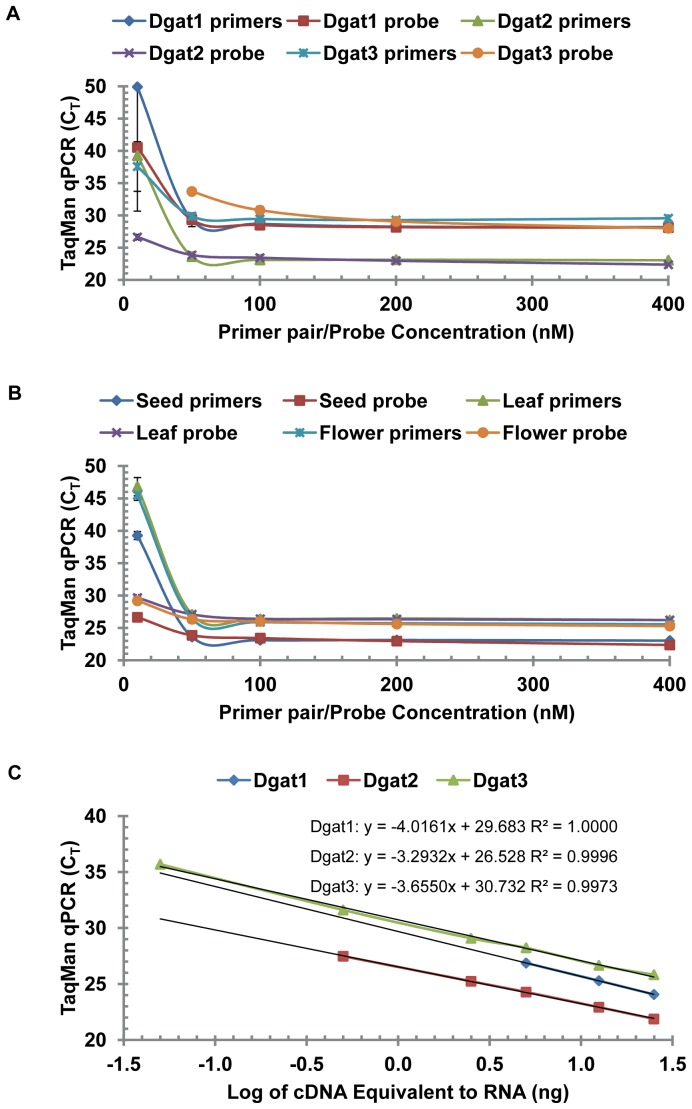
TaqMan qPCR optimization and efficiency for DGAT assay. (A) Optimization for DGAT1, DGAT2 and DGAT3 using RNA from tung seeds. (B) Optimization for DGAT2 using RNA from tung seeds, leaves and flowers. (C) TaqMan qPCR efficiency for DGAT assay. For qPCR optimization, TaqMan qPCR reactions contained 5 ng RNA-equivalent cDNA from tung seeds, leaves and flowers, various concentrations of the forward primer, reverse primer and TaqMan probe, and Absolute QPCR Mix. For qPCR efficiency, TaqMan qPCR reaction mixtures contained variable concentrations of RNA-equivalent cDNA from tung seeds, the optimized concentrations of each primer and probe (200 nM), and Absolute QPCR Mix. The results using RNA isolated from stage 4 of tree 1 are shown in the figure. The results using RNA from other stages of tung seeds, leaves and flowers are presented in Table 3.

TaqMan qPCR is often used for quantitative gene expression, which utilizes a fluorogenic single-stranded oligonucleotide probe that binds to specific DNA sequence between the two sequence-specific PCR primers, resulting in specific PCR products [40]. Optimization of TaqMan qPCR assay for DGAT family gene expression in tung tree tissues was performed first for better comparison of DGAT gene expression. The concentrations of TaqMan primers and probes were optimized using 5 ng RNA-equivalent cDNA from tung seeds, leaves and flowers. qPCR reactions contained 10–400 nM each of forward primer and reverse primer and a fixed probe concentration of 200 nM (for TaqMan primers optimization) or 10–400 nM of TaqMan probe and fixed primer pair concentrations of 200 nM (for TaqMan probe optimization). Figure 3 shows that the C_T_ value of qPCR amplification was much higher when the primer pair concentrations or the probes in the qPCR amplification mixtures were only 10 nM, but the C_T_ values were similar when higher concentrations of primer pairs and probes were used in the assays. Primer pair and probe concentrations of 100 nM saturated the TaqMan qPCR reactions using tung seed cDNA, for all three DGAT assays (Figure 3A). Similar qPCR results were obtained using cDNA from tung seeds, leaves and flowers (Figure 3B). These results indicated that 100 nM or above of the forward primer, reverse primer and TaqMan probe were optimal concentrations for TaqMan qPCR assay for quantitative DGAT gene expression in tung tree tissues. We thus used 200 nM for each of the primer and probe to evaluate qPCR efficiency for DGAT mRNA under variable template of RNA-equivalent cDNA (0.05–25 ng) from tung seeds, leaves and flowers. Semilog plots demonstrated excellent correlation between the log of cDNA equivalent to RNA in the qPCR assays and the TaqMan qPCR amplification (C_T_) (Figure 3C). TaqMan qPCR assays generated high correlation co-efficiency (r^2^>0.99 in most assays) and good amplification efficiency (Table 3). The qPCR amplification efficiencies for DGAT1, DGAT2 and DGAT3 in the three tissues were similar (96.13±14.25%, n = 21) (Table 3). TaqMan qPCR amplification efficiencies for DGAT gene expression were comparable to those for Rpl19b, Gapdh and Ubl reference gene expression in tung tree tissues [55].

**Table 3:** TaqMan qPCR efficiency for quantifying DGAT family mRNA in tung seeds, leaves and flowers.

Tung tissue	mRNA	Slope	Y-Intercept	Correlation Co –efficiency	Efficiency E = [10(1/−S)−1]*100 (%)
Seed (week 2)	DGAT1	−3.658	30.241	0.9981	87
	DGAT2	−3.242	27.786	0.9888	103
	DGAT3	−3.765	27.970	0.9989	85
Seed (week 4)	DGAT1	−2.839	29.089	0.9597	125
	DGAT2	−3.503	26.464	0.9998	93
	DGAT3	−3.596	28.096	0.9894	90
Seed (week 6)	DGAT1	−3.359	30.663	0.9944	99
	DGAT2	−3.389	25.609	0.9991	97
	DGAT3	−3.708	31.834	0.9936	86
Seed (week 8)	DGAT1	−3.796	28.925	0.9748	83
	DGAT2	−3.580	25.451	0.9981	90
	DGAT3	−3.807	30.150	0.9930	83
Seed (week 10)	DGAT1	−3.336	29.285	0.9947	100
	DGAT2	−3.395	25.555	0.9989	97
	DGAT3	−3.774	30.136	0.9984	84
Leaf	DGAT1	−2.807	28.904	0.9943	127
	DGAT2	−3.478	29.249	0.9985	94
	DGAT3	−3.685	26.967	0.9981	87
Flower	DGAT1	−3.101	28.644	0.9984	110
	DGAT2	−3.098	28.099	1.0000	110
	DGAT3	−3.884	26.369	0.9572	81

TaqMan qPCR reaction mixtures contained variable amounts of RNA-equivalent cDNAs from tung seed (2.5, 5, 12.5 and 25 ng), the optimized concentrations of each primer and probe (200 nM) and Absolute QPCR Mix. The amplification efficiency of qPCR assay is calculated according to the equation *E* = (10^−1/slope^ −1)×100.

### DGAT Gene Expression in Developing Tung Seeds, Leaves and Flowers

**Figure 4:**
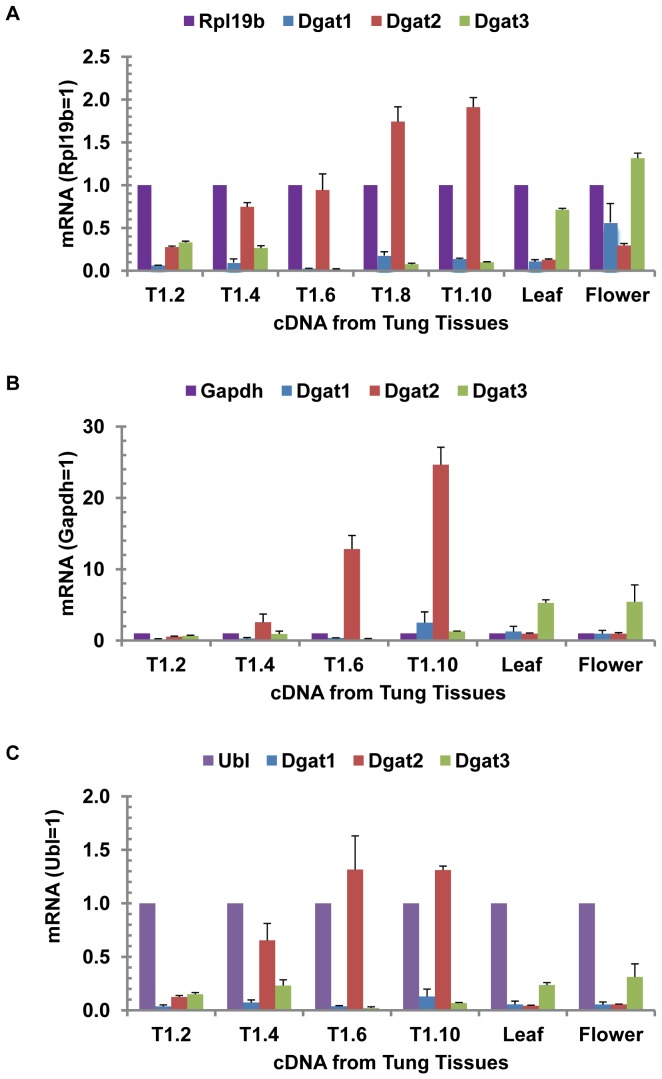
Relative expression of DGAT genes in tung seeds, leaves and flowers. TaqMan qPCR reaction mixtures contained variable amounts of RNA-equivalent cDNAs (2.5, 5, 12.5 and 25 ng) from tung tissues and the optimized concentrations of each primer and probe (200 nM). The mean and standard deviation of mRNA expression levels calculated from the four concentrations of DNA templates are presented. The results are representative data from three experiments. (A) Rpl19b as the reference mRNA. (B) Gapdh as the reference mRNA. (C) Ubl as the reference mRNA. T1 represents RNA extracted from tung tree 1. The number after T1 represents the week when tung seeds were collected.

Upon establishment of reliable TaqMan qPCR assay conditions, with optimal concentrations of primers and probe and excellent qPCR efficiency, this method was used to evaluate the expression profiles of the three tung DGAT genes, using well-characterized Rpl19b, Gapdh and Ubl as internal reference controls [55], [56]. Figure 4A shows that DGAT2 was the most abundant mRNA in tung seeds from all oil-accumulating stages, whereas DGAT3 was the most abundant mRNA in tung leaves, flowers and immature seeds prior to oil accumulation. Tung tissues with the highest expression levels of DGAT1, DGAT2 and DGAT3 as compared to the reference mRNA Rpl19b were flower, seed and flower, respectively (Figure 4A). Similar expression profiles of DGAT genes in tung tissues were observed using two other reference genes Gapdh (Figure 4B) and Ubl (Figure 4C).

### Relative Expression of DGAT Genes among Tung Trees

**Figure 5:**
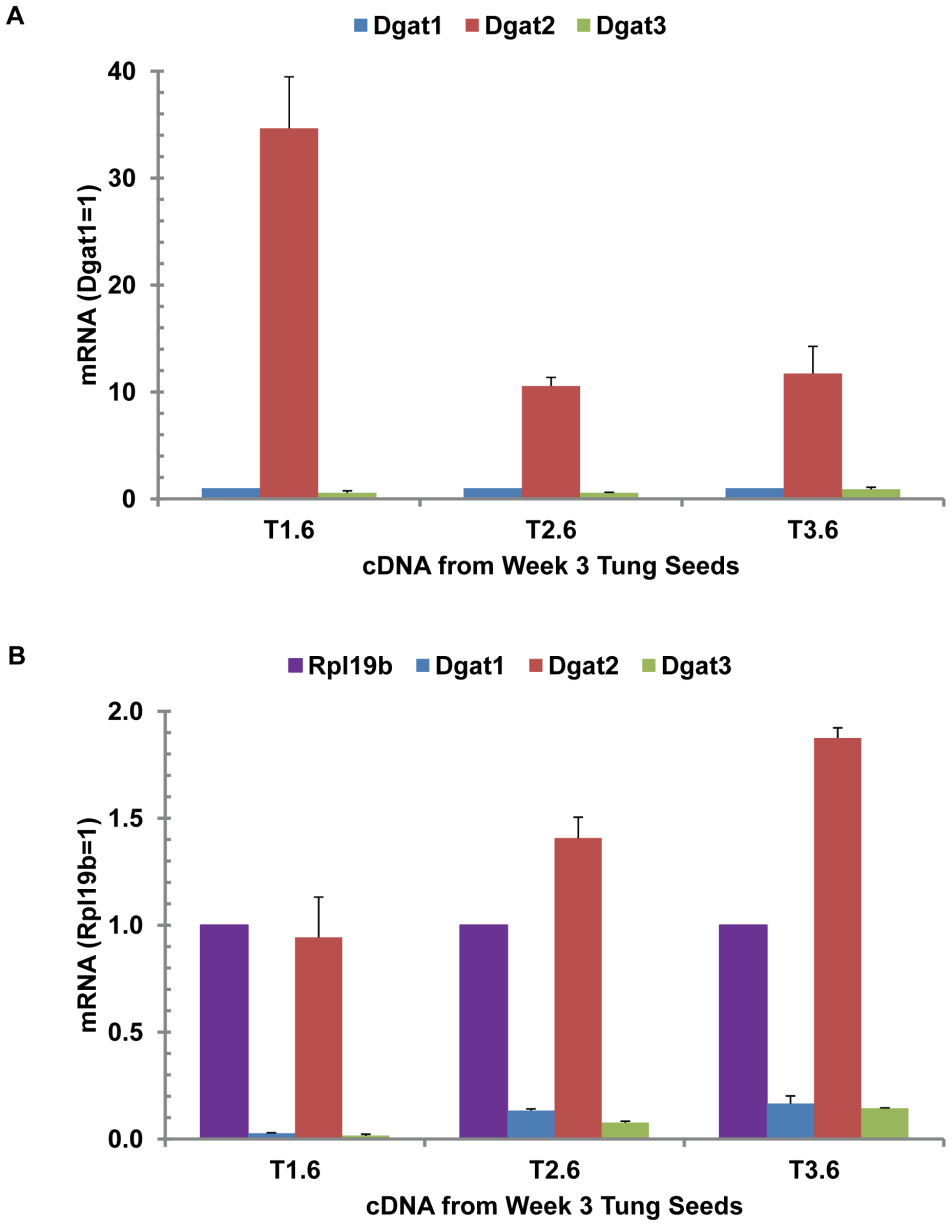
Relative expression of DGAT genes in tung seeds of three individual trees. TaqMan qPCR reaction mixtures contained variable amounts of RNA-equivalent cDNAs (2.5, 5, 12.5 and 25 ng) from week 6 seeds of three individual tung trees and the optimized concentrations of each primer and probe (200 nM). The mean and standard deviation of mRNA expression levels calculated from the four concentrations of DNA templates are presented using Rpl19b as the reference mRNA. (A) Expression relative to DGAT1. (B) Expression relative to Rpl19b. T1, T2 and T3 represent RNA extracted from tung trees 1, 2 and 3. The number 6 after T1, T2, and T3 represents tung seeds collected at week 6.

TaqMan qPCR was used to detect DGAT mRNA levels in oil-rich seeds from three individual tung trees to understand the variation of DGAT gene expression among the trees. DGAT2 was the most abundant mRNA in the seeds from all three trees, with over 10- to 30-fold higher mRNA levels compared to DGAT1 or DGAT3 (Figure 5A). The variations among the three trees were very significant, probably due to differences in timing of flowering among the trees. DGAT2 mRNA levels were equal to or greater than the housekeeping gene Rpl19b in stage 6 of tung seeds (Figure 5B).

### SYBR Green qPCR Analysis of DGAT Gene Expression in Tung Tissues

**Figure 6:**
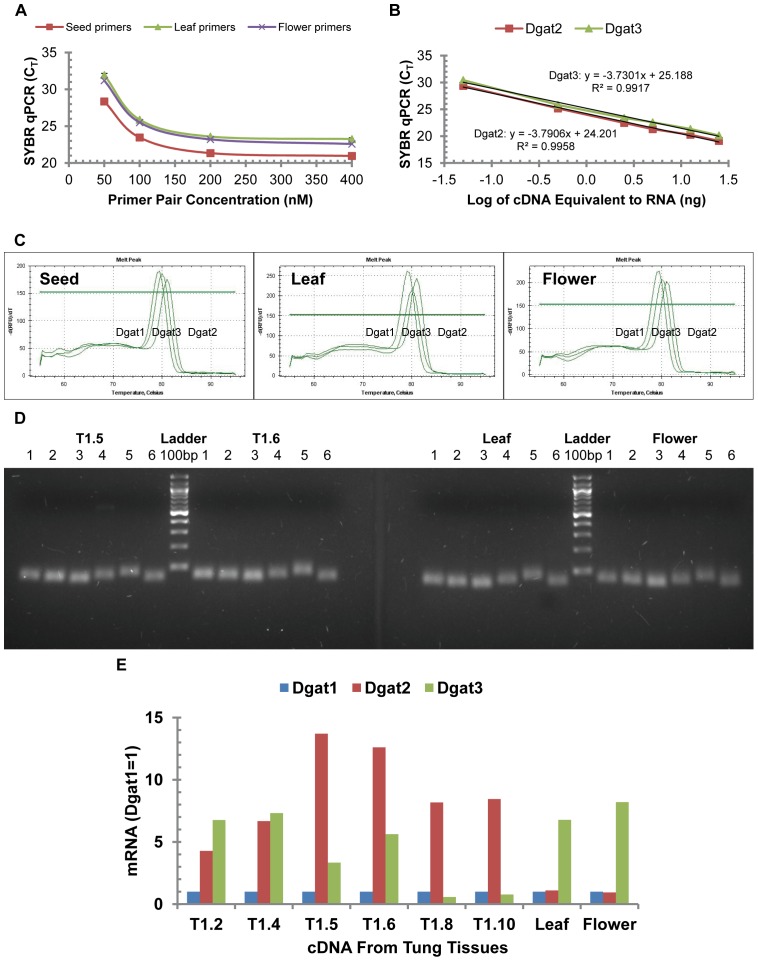
SYBR Green qPCR confirmation of DGAT gene expression in tung tree tissues. (A) SYBR Green qPCR primer optimization for DGAT2. The qPCR reaction mixtures contained 5 ng RNA-equivalent cDNA from tung seeds, leaves and flowers, various concentrations of the forward primer and reverse primer and iQ SYBR Green Supermix. (B) SYBR Green qPCR efficiency. The qPCR reaction mixtures contained variable concentrations of RNA-equivalent cDNA from tung seeds, the optimized concentrations of each primer and probe (200 nM) and iQ SYBR Green Supermix. The results using RNA isolated from stage 4 of tree 1 are shown in the figure. (C) Melt curve analysis of SYBR Green qPCR assays. The qPCR reactions contained 5 ng RNA-equivalent cDNA from tung seeds (left), leaves (middle) and flowers (right), the forward primer, reverse primer and iQ SYBR Green Supermix. (D) Gel electrophoresis of SYBR Green qPCR amplification products. The qPCR products after 40 cycles of amplification were separated by electrophoresis with 3% agarose gel at 100 V for 30 min. T1.5 and T1.6 represent RNA extracted from seed stages 5 and 6 of tung tree 1. Similar gel electrophoresis results were obtained using other stages of tung seeds (data not shown). Lanes 1–6 represent qPCR products for Rpl19b, Gapdh, Ubl, Dgat1, Dgat2, and Dgat3, respectively. The lane “Ladder 100 bp” represents the 100 bp DNA ladders with the lowest band being 100 bp. (E) Relative DGAT mRNA levels in tung seeds, leaves and flowers. The qPCR reaction mixtures contained 5 ng RNA-equivalent cDNA from tung seeds, leaves and flowers and the optimized concentrations of each primer (200 nM). The means of two determinations of DGAT mRNA levels are presented. T1 represents RNA extracted from tung tree 1. The number after T1 represents the week when tung seeds were collected.

SYBR Green qPCR is the other widely used method for quantitative gene expression. SYBR Green dye intercalates into double-stranded DNA to monitor the amplification of target gene specifically initiated by DNA-specific primers. Both TaqMan and SYBR Green qPCR assays are reliable for quantitative gene expression in tung tree tissues [42] and animal cells [43]. Therefore, SYBR Green qPCR was used to confirm the conclusion drawn from TaqMan qPCR as described in Figures 4 and 5, which showed that DGAT2 gene was highly expressed in tung seeds whereas DGAT3 gene was highly expressed in tung leaves, flowers and immature seeds. Figure 6A shows that primer concentrations at 200 nM almost saturated SYBR Green qPCR assays. Under these primer concentrations, SYBR Green qPCR amplification efficiencies were similar between DGAG genes (Figure 6B) and similar to those of the TaqMan qPCR results (Figure 6B vs. Figure 3C) as well as those of the three reference genes [55].

One of the potential drawbacks of SYBR Green qPCR is that the dye binds to any double-stranded DNA non-specifically which may generate false positive signals if nonspecific products or primer-dimers are present in the assay. The specificity of SYBR Green qPCR amplification can be evaluated by melt curve analysis and gel electrophoresis. Melt curve analysis shows that each DGAT gene-specific qPCR resulted in a single peak of PCR amplification signal using total cDNA from seeds, leaves and flowers (Figure 6C), which were similar to those of the three reference genes [55]. Agarose gel electrophoresis shows that each DGAT as well as the reference gene-specific qPCR resulted in a single DNA fragment with predicted size of the amplicon (Figure 6D vs. Table 1). Both analyses indicated that SYBR Green qPCR assays were reliable for evaluating DGAT family gene expression under the optimized assay condition.

Therefore, DGAT family gene expression in tung tree tissues was evaluated by the optimized and verified SYBR Green qPCR assay. The results show that DGAT2 mRNA levels were several folds higher than DGAT1 in all stages of developing tung seeds (Figure 6E). Both DGAT1 and DGAT2 mRNA levels were similarly low in leaves and flowers (Figure 6E). DGAT2 mRNA levels were the highest in the middle (T1.5 and T1.6) and later stages (T1.8 and T1.10) of developing tung seeds, which were over 10-fold higher than DGAT1 mRNA levels in seed stages 5 and 6 (Figure 6E). DGAT3 mRNA levels were several folds higher than DGAT1 and DGAT2 in leaves and flowers, and several folds higher than DGAT1 in immature seeds (Figure 6E). SBYR Green qPCR results were in general agreement with TaqMan qPCR. Both qPCR assays show that DGAT2 was the most expressed DGAT gene in tung seeds and DGAT3 was the most expressed DGAT gene in leaves, flowers and immature seeds. There were some fold differences between these two qPCR assays. These differences in relative quantitation of DGAT gene expression might be due to the different chemistries of the two qPCR assays utilized.

### Northern Blotting Confirmation of DGAT2 as the Major DGAT mRNA in Tung Seeds

**Figure 7:**
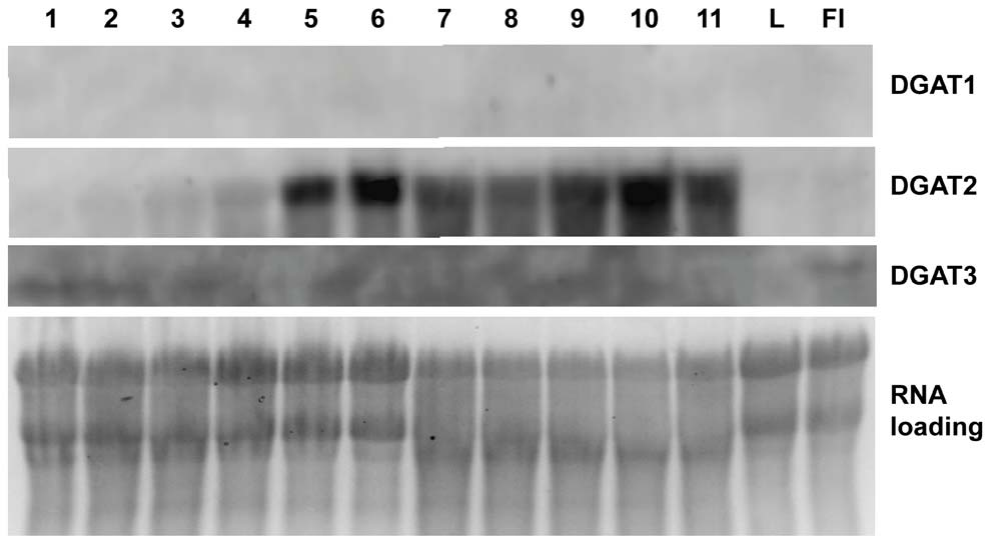
Northern blotting of DGAT1, DGAT2 and DGAT3 mRNA in tung tree tissues. Total RNA was extracted from tung leaves, flowers and developing seeds. Total RNA (15 µg RNA/lane) was blotted and probed as described in “Materials and Methods”. The DGAT1, DGAT2 and DGAT3 blots were developed for 10 min, 20 min and 50 min, respectively. Labels above the panels: 1–11-RNA isolated from tung seeds collected at week 1–11; L-RNA from leaves; Fl-RNA from flowers.

Both TaqMan and SYBR Green qPCR results presented above firmly demonstrated that DGAT2 was the most abundant mRNA of the DGAT family in developing tung seeds. Northern blotting was used to confirm the qPCR data. Figure 7 shows that DGAT2 mRNA was gradually increased in the first four weeks during seed development, but was dramatically up-regulated after week 5 and peaked at week 6, followed by a slight decline and a second peak at week 10. In contrast, DGAT1 mRNA was barely detected under identical experimental conditions but was weakly detected after overexposure of the blot (Figure 7). DGAT3 expression remained below the threshold of detection in all seed RNA samples under the same exposure time (data not shown), but was detected after overexposure in the flowers. Neither DGAT1 nor DGAT2 was clearly detectable in leaves or flowers under the same blotting conditions (Figure 7).

### Immunoblotting of DGAT2 in Developing Tung Seeds

**Figure 8:**
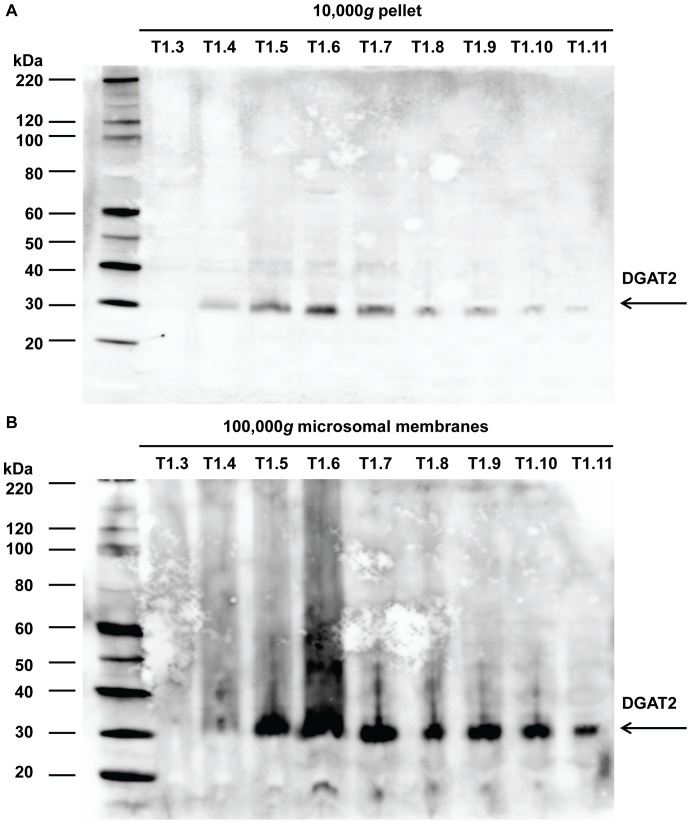
Immunoblotting of DGAT2 protein in developing tung seeds. The 10,000(A) and 100,000 g microsomal membranes (B) were prepared from tung seeds by differential centrifugation. DGAT2 protein was detected by immunoblotting using affinity-purified rDGAT2 antibodies. T1 represents protein samples extracted from seeds of tung tree 1. The number after T1 represents the week when tung seeds were collected.

Immunoblotting was used to confirm the expression profile of DGAT2 gene in developing tung seeds. Anti-rDGAT2 was raised against the recombinant MBP-DGAT2 (rDGAT2) in rabbits and affinity-purified against the purified antigen. These antibodies were used to detect endogenous DGAT2 in the 10,000 g pellet and 100,000 g microsomal membranes isolated from developing tung seeds. Immunoblotting detected a single protein band approximately corresponding to the size of endogenous DGAT2 protein (expected MW 36.7 kDa) in both fractions, but DGAT2 protein was highly enriched in the microsomal membranes (Figure. 8A vs. 8B). DGAT2 protein was detectable in week 4 seeds, rapidly increased between weeks 5 and 6 and decreased after week 7 (Figure 8). DGAT2 was undetectable in the 100,000 *g* supernatant under the same immunoblotting conditions (data not shown), suggesting that the levels of DGAT2 in the lipid-enriched soluble fraction were probably too low to be detected under the experimental conditions. DGAT2 protein levels in tung tree leaves and flowers were not analyzed by immunoblotting using the DGAT2 antibodies. The levels of DGAT2 in the leaves and flowers are probably too low to be detected since only low levels of DGAT2 mRNA were detected by qPCR (Figures 4 and 6) and undetectable by Northern Blot in these tissues (Figure 7).

### Oil Accumulation in Developing Tung Seeds

To correlated DGAT expression data with lipid accumulation, oil profiles in tung seeds were determined in lipid extracts after heat extraction of the oils from seeds collected at 11 weekly stages. Lipid profiles show that α-eleostearic acid (18∶3), the main component of tung oil, accumulated starting at approximately week 2, rapidly increased between weeks 3–5, and reached a plateau after week 7 (Figure 8).

## Discussion

Diacylglycerol acyltransferases (DGATs) are responsible for the final and rate-limiting step of TAG biosynthesis in eukaryotic organisms [32]. Plants and animals deficient in DGATs accumulate less TAG [34], [64], [65]. Animals with reduced DGAT activity resist to diet-induced obesity [65], [66], lack milk production [65] and link to a congenital diarrheal disorder [67]. Over-expression of the DGAT enzymes increases TAG content in plants [28], [68]–[73], animals [74]–[77] and yeast [78]. Therefore, understanding the roles of DGATs will help to create transgenic plants and microbes with value-added properties and provide information for therapeutic intervention for obesity and related diseases.

It is generally accepted that DGATs are divided into DGAT1 and DGAT2 subfamilies [16]. Phylogenetic analysis and multiple sequence alignment classify 117 DGAT protein sequences from 70 organisms into DGAT1 and DGAT2 subfamilies [33]. Multiple sequence alignment has shown that DGAT1s and DGAT2s have 41 and 16 completely conserved amino acid residues, respectively, which are mostly located at the carboxyl termini of DGATs [33]. However, recent studies show that more than two forms of DGATs are present in a number of species including yeast (*Rhodotorula glutinis*) [GenBank:DG315417.1] [79], *Arabidopsis thaliana* [GenBank:AAN31909.1] [80], Burning Bush (*Euonymus alatus*) [GenBank:GU594061.1] [28], peanut (*Arachis hypogaea*), [GenBank:AY875644.1] [29], castor bean (*Ricinus communis*) [GenBank:XP_002519339.1] and various other plant species. Phylogenetic analysis and multiple sequence alignment indicate that these sequences are different from DGAT1s and DGAT2s. In fact, none of the completely conserved residues in DGAT1s (41 residues) and DGAT2s (16 residues) is aligned with these newly identified DGATs [33].

Many genes from the tung oil biosynthetic pathway have been cloned in our laboratories in recent years. We have identified and cloned genes for type-1 and type-2 DGAT proteins from tung [16] and determined the sequence for type-3 DGAT in this study. Biochemical studies suggest that total DGAT enzyme activity is a rate-limiting step during TAG synthesis in oilseeds [81]. However, direct determination of the role of specific DGAT isoforms in oilseed tissues is complicated by the existence of multiple forms of DGAT isoforms [27]–[29], [33], [64], [80]. It is important to determine which isoform(s) of DGAT is expressed in developing seeds to guide successful metabolic engineering efforts. The identification of potential transgenic targets could be accomplished at least in part by analyzing the expression patterns for all known isoforms of the genes predicted to be involved in tung oil biosynthesis. In this study, we identified the putative soluble DGAT3 gene and used real-time qPCR for quantitative DGAT family gene expression analysis in tung seeds, leaves and flowers, followed by confirmation with northern and western blotting techniques.

Tung DGAT3 has only 278 amino acid residues (Figure 1). It is the smallest among the three tung DGAT isoforms and the smallest among 27 DGAT3 sequences from 19 plant species (Table 2). Tung DGAT3 has approximately 31% hydrophobic residues, compared to 41% and 43% for DGAT1 and DGAT2, respectively [33]. Tung DGAT3 protein is highly similar to DGAT3s from other species and contained all 11 residues conserved amongst this protein class (Figure 2). The full-length sequence of tung DGAT3 protein aligns well with the amino and carboxyl termini of the other plant DGAT3s, which are modestly conserved among the 27 DGAT3s from 19 plant species (Figure S2). Tung DGAT3 does not align with the completely conserved residues in DGAT1s (41 residues) and DGAT2s (16 residues) [33] and only four residues are completely conserved among the three tung DGAT proteins (Figure 1). These data clearly suggest that the cloned DGAT3 from tung tree is a full-length DGAT3. Unlike DGAT1s and DGAT2s, DGAT3 homologues are not present in any animal species.

There are significant differences among the 27 DGAT3 protein sequences from 19 plant species. Most notably, a short insertion before the first completely conserved serine residue and two long insertions between this conserved serine residue and the second completely conserved cysteine residue are presented in DGAT3s of cereals including rice, barley, corn and sorghum (Figure S2). DGAT3 sequences among the species producing unusual fatty acids such as tung tree and castor bean [5], [82] have variable lengths of insertions after the last completely conserved valine residue (Figure S2). How the divergences between individual or subsets of DGAT3 enzymes contribute to the production of unique unusual fatty acids in plants requires future investigation.

This study has provided complete expression profiles of all known DGAT genes in tung tissues and shown that all three isoforms of DGAT family genes are expressed in developing seeds, leaves and flowers. It is still possible that the tung tree genome contains other forms of DGAT-like proteins. The major findings of the quantitative gene expression analyses are that DGAT2 is firmly established as the most strongly expressed DGAT mRNA in tung seeds (Figures 4–6) and that DGAT2 mRNA and protein expression profiles are well-coordinated with the oil and α-eleostearic acid accumulation profiles in developing tung seeds (Figures 4–8 vs. 9). TaqMan qPCR assay has demonstrated that DGAT2 mRNA is the most abundant DGAT mRNA in the seeds from all oil-accumulating stages with over 10- to 30-fold higher levels of expression than DGAT1, whereas DGAT3 is the most abundant mRNA in leaves, flowers and immature seeds prior to the onset of oil deposition (Figures 4–5). The DGAT2 expression profile revealed by TaqMan qPCR is confirmed by results from SYBR Green qPCR (Figure 6), northern blotting (Figure 7) and western blotting (Figure 8). The qPCR and northern blot data shown here also correlate very well with previous northern blot data produced using a different set of developing tung seed samples [16]. More direct evidence for correlating DGAT gene product levels to oil production in tung trees will come from enzymatic assays using protein extracts from developing tung seeds. This approach will also help to address possible differences in protein translation efficiency and steady state protein levels in the native tung seed environment. Nonetheless, the results presented in this report, together with the findings presented by Shockey et al. [16], suggest that DGAT2 is probably the major contributor to tung oil biosynthesis in the seeds. The expression profile of DGAT3 gene in tung tissues suggest that DGAT3 enzyme is probably not significantly involved in tung oil biosynthesis in seeds; however, it may be involved in TAG metabolism in other tung tissues.

**Figure 9:**
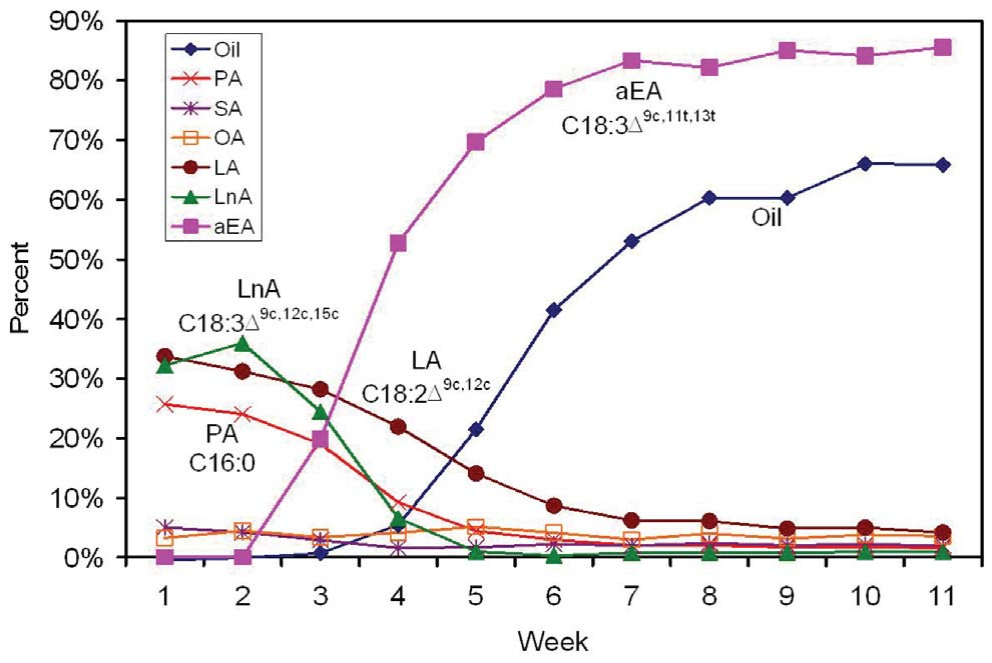
Oil accumulation in developing tung seeds. Lipids were extracted from developing tung seeds collected weekly. Total seed oil content was determined by weight. Seed lipid composition was determined by gas chromatography after converting all lipids to methyl esters. The peaks corresponding to lipid fatty acids were identified by comparing to residence times for fatty acid methyl ester standards.

Finally, we have developed anti-tung DGAT2 antibodies, which detect endogenous DGAT2 protein in developing tung seeds. The tung DGAT2 protein expression profile is well-correlated with its mRNA levels and tung oil and α-eleostearic acid levels in developing seeds (Figures 8 vs. 9). These data provide additional support for the predicted dominant role of DGAT2 in driving these processes in developing tung seeds, and for the observation that tung DGAT2 expression is primarily regulated at the transcriptional level (Figures 4–6 and [16]). Significant progress has been made in the identification of DGAT genes from numerous organisms [33], but the studies of DGAT proteins are limited. It is difficult to purify DGAT proteins from any source [58], [83]–[85], probably because these proteins are integral membrane proteins containing approximately 40% hydrophobic amino acid residues overall [33]. DGAT genes have been cloned for 15 years [16], [27], [86], [87]; however, no high-titer anti-DGAT antibodies for relevant research were reported until now. We have developed reliable protocols for recombinant DGAT protein expression and purification [58], [83]. In this study, we have raised DGAT2-specific antibodies using recombinant DGAT2 protein. Western blotting shows that DGAT2 protein is maximally expressed in seeds actively producing tung oil. To our knowledge, this is the first convincing demonstration of immunological detection of endogenous DGAT2 protein in any plant. A recent report has shown that DGAT2 of *Arabidopsis* is identified by rabbit polyclonal antibodies raised against a peptide corresponding to amino acid residues 191–260 of human DGAT2 [88]. The cross-reactivity could be explained by the fact that the epitope region of human DGAT2 contains highly conserved motifs with 6 completely conserved residues among 54 diverse organisms including human and *Arabidopsis* (PR block in motif2, GGE block in motif 3, and R of RGFA block in motif 4 [33]). However, more experiments need to be done to confirm the specificity of the immune cross-reactivity between plant proteins and human antibodies. The availability of the DGAT2-specific antibodies should greatly facilitate protein-focused research approaches.

## Conclusions and Future Research

Gene cloning and real-time qPCR, coupled with Southern, northern and western blotting techniques, have demonstrated that: 1) a copy of DGAT3 gene is present in tung tree genome; 2) all three isoforms of DGAT family genes are expressed in developing seeds, leaves and flowers; 3) DGAT2 is firmly established as the major DGAT mRNA in tung seeds; 4) DGAT3 gene likely plays a significant role in TAG metabolism in tung leaves, flowers and immature seeds prior to active tung oil biosynthesis; and 5) endogenous DGAT2 protein is detected in developing tung seeds using DGAT2 protein-specific antibodies raised against recombinant DGAT2 protein. The levels of DGAT2 mRNA and protein are well-correlated with the profiles of oil accumulation in developing tung seeds. These results suggest that DGAT2 protein is probably the major contributor to tung oil biosynthesis in the seeds. The results described in this report should facilitate future research in areas such as understanding the contributions of each DGAT isoform in TAG metabolism, identification of additional enzymatic components of the tung oil biosynthetic apparatus, and testing the potential of using genes like tung DGAT2 to produce industrial oils in engineered oilseed crops.

## Supporting Information

Figure S1 **Nucleotide sequence alignment of the three tung DGATs.** DGAT sequence name is on the left of alignment followed by the start of the nucleotide sequence of each DGAT. The numbers at the top of the alignment are the positions of the multiple sequence alignment. The letters at the bottom of the alignment are the consensus nucleotides. Nucleotides in red on yellow represent those conserved in all three DGAT sequences at a given position, whereas those in black on blue represent nucleotides conserved in two of the three sequences at a given position. The underlined nucleotides represent the forward primers, TaqMan probes and the complementary sequences of the reverse primers for the three DGAT qPCR assays.
(PDF)

Figure S2 **Identification of amino acid residues and sequence motifs conserved in DGAT3s.** Each DGAT sequence name is on the left of the alignment followed by the position of amino acid residue of DGAT protein sequence in the alignment. The letters at the bottom of the alignment are the consensus residues. Color codes for amino acid residues are as follows: 1) red on yellow: consensus residue derived from a completely conserved residue at a given position; 2) blue on cyan: consensus residue derived from the occurrence of greater than 50% of a single residue at a given position; 3) black on white: non-similar residues. The abbreviations of the organisms are: Ah, *Arachis hypogaea* (peanut); At, *Arabidopsis thaliana*; Bd, *Brachypodium distachyon;* Gm, *Glycine max* (soybean); Hv, *Hordeum vulgare (barley)*; Lj, *Lotus japonicas*; Mt, *Medicago truncatula*; Os, *Oryza sativa* (rice); Pg, *Picea glauca* (white spruce); Pp, *Physcomitrella patens*; Ps, *Picea sitchensis* (sitka spruce); Pt, *Populus trichocarpa*; Rc, *Ricinus communis* (caster bean); Sb, *Sorghum bicolor* (sorghum); Sl, *Solanum lycopersicum* (tomato); Sm, *Selaginella moellendorffii*; Vf, *Vernicia fordii* (tung tree); Vv, *Vitis vinifera* (grape); Zm, *Zea mays* (corn).
(PDF)
