# Supplementary figures and images for: Developmental Regulation of Diacylglycerol Acyltransferase Family Gene Expression in Tung Tree Tissues
Source: PLoS One. 2013 Oct 11;8(10):e76946. doi: 10.1371/journal.pone.0076946 (PMC3795650; doi:10.1371/journal.pone.0076946)

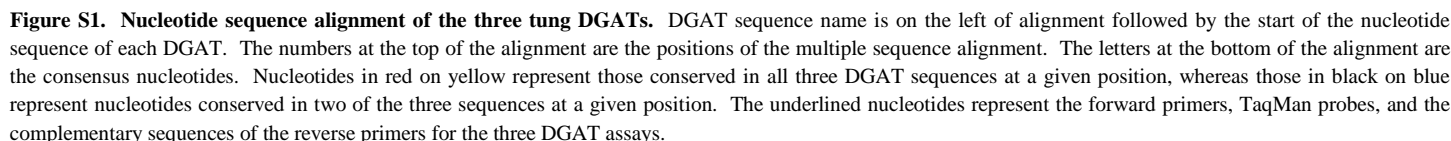

Supplement: Figure S1 — Nucleotide sequence alignment of the three tung DGATs. DGAT sequence name is on the left of alignment followed by the start of the nucleotide sequence of each DGAT. The numbers at the top of the alignment are the positions of the multiple sequence alignment. The letters at the bottom of the alignment are the consensus nucleotides. Nucleotides in red on yellow represent those conserved in all three DGAT sequences at a given position, whereas those in black on blue represent nucleotides conserved in two of the three sequences at a given position. The underlined nucleotides represent the forward primers, TaqMan probes and the complementary sequences of the reverse primers for the three DGAT qPCR assays. (PDF) [file pone.0076946.s001.pdf]
