# Supplementary material for: Developmental Regulation of Diacylglycerol Acyltransferase Family Gene Expression in Tung Tree Tissues
Source: PLoS One. 2013 Oct 11;8(10):e76946. doi: 10.1371/journal.pone.0076946 (PMC3795650; doi:10.1371/journal.pone.0076946)
Supplement: Figure S2 — Identification of amino acid residues and sequence motifs conserved in DGAT3s. Each DGAT sequence name is on the left of the alignment followed by the position of amino acid residue of DGAT protein sequence in the alignment. The letters at the bottom of the alignment are the consensus residues. Color codes for amino acid residues are as follows: 1) red on yellow: consensus residue derived from a completely conserved residue at a given position; 2) blue on cyan: consensus residue derived from the occurrence of greater than 50% of a single residue at a given position; 3) black on white: non-similar residues. The abbreviations of the organisms are: Ah, Arachis hypogaea (peanut); At, Arabidopsis thaliana; Bd, Brachypodium distachyon; Gm, Glycine max (soybean); Hv, Hordeum vulgare (barley); Lj, Lotus japonicas; Mt, Medicago truncatula; Os, Oryza sativa (rice); Pg, Picea glauca (white spruce); Pp, Physcomitrella patens; Ps, Picea sitchensis (sitka spruce); Pt, Populus trichocarpa; Rc, Ricinus communis (caster bean); Sb, Sorghum bicolor (sorghum); Sl, Solanum lycopersicum (tomato); Sm, Selaginella moellendorffii; Vf, Vernicia fordii (tung tree); Vv, Vitis vinifera (grape); Zm, Zea mays (corn). (PDF) [file pone.0076946.s002.pdf]

```
                                 1                                                                                                  100
AhDGAT3-AY875644.1        (1) ----------------------------------------------------------------------------------------------------
GmDGAT3-XP_003542403      (1) ----------------------------------------------------------------------------------------------------
LjDGAT3-AFK37850          (1) ----------------------------------------------------------------------------------------------------
MtDGAT3-ACJ86204          (1) ----------------------------------------------------------------------------------------------------
AtDGAT3-AAD49767.1        (1) ----------------------------------------------------------------------------------------------------
AtDGAT3-NP_175264         (1) ----------------------------------------------------------------------------------------------------
AtDGAT3-XP_002891423      (1) ----------------------------------------------------------------------------------------------------
VvDGAT3-CBI26023          (1) ----------------------------------------------------------------------------------------------------
VvDGAT3-XP_002269582      (1) ----------------------------------------------------------------------------------------------------
PtDGAT3-XP_002314335      (1) ----------------------------------------------------------------------------------------------------
RcDGAT3-XP_002519339.1    (1) ----------------------------------------------------------------------------------------------------
VfDGAT3                   (1) ----------------------------------------------------------------------------------------------------
BdDGAT3-XP_003568982.1    (1) ----------------------------------------------------------------------------------------------------
HvDGAT3-BAJ97917          (1) ----------------------------------------------------------------------------------------------------
OsDGAT3-EAY96477          (1) ----------------------------------------------------------------------------------------------------
OsDGAT3-NP_001054585      (1) ----------------------------------------------------------------------------------------------------
SbDGAT3-XP_002439241      (1) ----------------------------------------------------------------------------------------------------
ZmDGAT3-ACR36974          (1) ----------------------------------------------------------------------------------------------------
ZmDGAT3-NP_001183501      (1) ----------------------------------------------------------------------------------------------------
PpDGAT3-XP_001764081      (1) MTIATTGQKKRSKTHLVLSPIRKMVSPGSPRSYSSAEVIKSKPKMTDAALSTPSHRVEPFPNLLGHPCHQLTLGSGLVPSLVGSSLFDVVYDFGIHAVQL
SmDGAT3-XP_002964239      (1) ----------------------------------------------------------------------------------------------------
PgDGAT3-BT109069          (1) ----------------------------------------------------------------------------------------------------
PsDGAT3-ABR16961          (1) ----------------------------------------------------------------------------------------------------
PgDGAT3-BT111835          (1) ----------------------------------------------------------------------------------------------------
PsDGAT3-ABK23206          (1) ----------------------------------------------------------------------------------------------------
PtDGAT3-XP_002301551      (1) ----------------------------------------------------------------------------------------------------
SlDGAT3-AK247265          (1) ----------------------------------------------------------------------------------------------------
Consensus                 (1)

                              101                                                                                                 200
AhDGAT3-AY875644.1        (1) ---------------------------------------MEVSGAVLRNVTCPSFSVHVSSRRRGGDSCVT----VP------------VRMRKKAVVRC
GmDGAT3-XP_003542403      (1) ---------------------------------------MEISGSVLRQLSYVSGYGTPTRSRG-------------------------VASRVGLRMGT
LjDGAT3-AFK37850          (1) ---------------------------------------MDVSGTILRQLTYVTGAGTNAHSRGA----------RV------------WAARPTARVVM
MtDGAT3-ACJ86204          (1) ---------------------------------------MEVSGTVLRHTNYIFGVGTHTRPHG--------------------------VVPRRVRMSM
AtDGAT3-AAD49767.1        (1) ---------------------------------------MEVSGVVLRQIPCVSSGSVAGLRLVSEFSGNTRTVGFR--------------TRRFRGIVC
AtDGAT3-NP_175264         (1) ----------------------------------------------------------------------------------------------------
AtDGAT3-XP_002891423      (1) ---------------------------------------MEVSGVVLRQIPCVSSGSVACLRLVSEFSGNTRTVGFR--------------TKKFRGIVC
VvDGAT3-CBI26023          (1) ---------------------------------------MEVSGVVFRQVPFFSGAGIDTQSSKSSFSGVSVDSGNRISAFSELRLLGSRDSRVAVRPRK
VvDGAT3-XP_002269582      (1) ---------------------------------------MEVSGVVFRQVPFFSGAGIDTQSSKSSFSGVSVDSGNRISAFSELRLLGSRDSRVAVRPRK
PtDGAT3-XP_002314335      (1) ----------------------------------------------------------------------------------------------------
RcDGAT3-XP_002519339.1    (1) --------------------------------------------MEVSGLGCFSSAATPSLCGAVDSGGVSSLRPRK---------------AFHRVSDS
VfDGAT3                   (1) ---------------------------------------MELSGVALRQLPCFSSRIVNS--S---------------------------EFRDKGHLQC
BdDGAT3-XP_003568982.1    (1) ---------------------------------------MEFTGAAALRRSLPAACPAVFS-RERRNRG------------------MPGRVSCVGRG-G
HvDGAT3-BAJ97917          (1) -------------------------------------MELTATGAAALRRSLASAYTVVP--RERR-RG------------------MPSRVSCVGRGGI
OsDGAT3-EAY96477          (1) ---------------------------------------MELTGAAIRGSLATAASPVVLRGRGRR---------------------CAARVSCVGRG--
OsDGAT3-NP_001054585      (1) ---------------------------------------MELTGAAIRGSLATAASPVVLRGRGRR---------------------CAARVSCVGRG--
SbDGAT3-XP_002439241      (1) ---------------------------------------MELTGAALRRYALPSASPVSARVGRRRR--------------------LPARVACVGGG--
ZmDGAT3-ACR36974          (1) ----------------------------------------------------------------------------------------------------
ZmDGAT3-NP_001183501      (1) ---------------------------------------MELTGAALRRYALPSASPVAARVGRRR---------------------RPARVACVGGG--
PpDGAT3-XP_001764081    (101) GSTWGDSSGARPMTLSEPLLLDSSIASLHVIHSLGESGLGEFDREDLRLNPVGGVFAMEVAVSGSRRAVERLQSLRGLGVSREFCGGVEVRGVVKRVEVC
SmDGAT3-XP_002964239      (1) ----------------------------------------------------------------------------------------------------
PgDGAT3-BT109069          (1) -------------------------------------METESAMETSARSGAMETTSCLSNESRGINTNYVAVGINR------------VSLSSNRQTVS
PsDGAT3-ABR16961          (1) -------------------------------------------MESAMSARSGAMTSCLSNESRGINTNYVAVGISR------------VSLSSNRQTVS
PgDGAT3-BT111835          (1) ---------------------------------------METESAISARNGAMETVGSLSN-----------I----------------VSLPSNRETVS
PsDGAT3-ABK23206          (1) -----------------------------------------MESAISARNG--AMVGSLSN-----------I----------------VSLSSNRETVS
PtDGAT3-XP_002301551      (1) --------------------------------------METARNIIPRPVLCFPGTRIATHRLSLPPNG-----------LGTGKCGVPFRSKRYDHRIL
SlDGAT3-AK247265          (1) -------------------------------------------------------------------------------------------------MET
Consensus               (101)                                        ME  G   R

                              201                                                                                                 300
AhDGAT3-AY875644.1       (46) CCGFSDSGHVQYYGDEK----KKENGT---------------------AMLSTKK--KLKMLKKRVL----------FDDL-QG-----N----LTWDAA
GmDGAT3-XP_003542403     (37) GSGFCDEGHLQYYQDTK----K---------------------------ILTPK--KLKLLKGFSK-------LGLASDP-EKLAMFHDLQQNLTSDAG
LjDGAT3-AFK37850         (40) GSGFSDEGHLQYYQDKK----KG--KP---------------------VVLTAKN--KVKLLKRVSKGMSLFDELGFALDP-NQRALLNDLQTNLTSDSG
MtDGAT3-ACJ86204         (36) GSGFHDEGHVQYYQDVK----KNT-EP---------------------VIISNKK--KIKLLKRFSKNVSQLPQLGFAQDP-N---LLDQLHQNLITEGG
AtDGAT3-AAD49767.1       (48) NNEFADKGHVNYYIEPT----RCGEEKEKV-------------KVMEKEKKALKK--KAKVLKSLSKNLDMFSSIGFGLDP-EA-GLVGEIQTKTISEAT
AtDGAT3-NP_175264         (1) ---------------------------------------------MEKEKKALKK--KAKVLKSLSKNLDMFSSIGFGLDP-EA-GLVGEIQTKTISEAT
AtDGAT3-XP_002891423     (48) NNEFADKGHMSYYIEPT----RCGEEKEKV-------------KVMEKEKKALKK--KEKVLKSLSKNLNMFSSIGFGLDP-EA-GLVGEIQTKTISEAT
VvDGAT3-CBI26023         (62) PSGFRDESHLKYYYESP----RCGAKK-------------------DKDKVTTKK--KSKLLKALSKDLSLFSDLGFGVDS-DE-GLFGEVKGKMISEAA
VvDGAT3-XP_002269582     (62) PSGFRDESHLKYYYESP----RCGAKK-------------------DKDKVTTKK--KSKLLKALSKDLSLFSDLGFGVDS-DE-GLFGEVKGKMISEAA
PtDGAT3-XP_002314335      (1) ----------------------------------------------------------------------------------------------------
RcDGAT3-XP_002519339.1   (42) CLGFRDNGHLQYYCQGG----FVRCGG--------------------GNKKSIKK--KLKLVKSLSEDFSMFPHNNA---------LLHQPQSISLQEAA
VfDGAT3                  (33) YNYYHGEARFVRCGPSS----LSGIKK-------------------EKESAMSKK--KLKLLKGLSKDLSVFFQIDP-----------DNSQAKLVAEAA
BdDGAT3-XP_003568982.1   (42) GVGFADEGHLRYYEAAP----R-------------------------------------KAVEAAARDLTKLRAMGLVAGD-PS-------KEKILSEAT
HvDGAT3-BAJ97917         (43) GGGFADEAHLRYYEAVP----R-------------------------------------KAVEAAARDLTKLRAMGLVAGD-PA-------KEKILSEAT
OsDGAT3-EAY96477         (39) GGGFGDEGHLRYYEAPP----R-------------------------------------KAVEAVARDLAKLRAMGIASGD-AA-------KEKVLSEAT
OsDGAT3-NP_001054585     (39) GGGFGDEGHLRYYEAPP----R-------------------------------------KAVEAVARDLAKLRPMGIAAGD-AA-------KEKVLSEAT
SbDGAT3-XP_002439241     (40) -GGFAEEGHLRYYEGAP----RR------------------------------------KAVEAVARDLGKLRAMGLVAGD-AA-------KEKVLSEAT
ZmDGAT3-ACR36974          (1) ----------------------------------------------------------------------------------------------------
ZmDGAT3-NP_001183501     (39) -G-FAEEGHLRYYEAAP----RR------------------------------------KAVEAVARDLGKLRAMGLVAGD-AA-------KEKVLSDAT
PpDGAT3-XP_001764081    (201) GAGFVDRGQESFYALRVRCGDKDKRVVG---------------RLARVQELVEAQRDLILSSRRALAQRSADVVRRLEVDDSLEIGVSDAVIDRIVQNVA
SmDGAT3-XP_002964239      (1) ----------------------------------------------------------------MSCSTRGQGSSGARRPP------ARAASPEVIRQLE
PgDGAT3-BT109069         (52) RLVFSDRNPVKSCGG------TAQPYCGMETKNKLEKQKLKHLSALSDGCLSEKKRMETKLLQLLSKDLSVLSAFPADANV-S---LAEQVRGEILSDAV
PsDGAT3-ABR16961         (46) RLVFSDRNPVKSCGG------TAQPYCGMK--NKLEKQKLKHLSALSDGCLSEKK--RMKLLQLLSKDLSVLSAFPADANV-S---LTEQVRGEILSDAV
PgDGAT3-BT111835         (35) RITFSDQSHLQYYGGME----TVKPYCGKK--NKLKKLKLKHLLPLNDRPLSDKR--KGNLLESLSKDLSALPTTSADANV-P---LTEQVRSQILSDAV
PsDGAT3-ABK23206         (31) RITFSDQSHLQYYG--G----MVKPYCGKK--NKLKKLKLKHLLPLNDRPLSDKR--KGNLLESLSKDLSALPTTSADANV-S---LTEQVRGQILSDAV
PtDGAT3-XP_002301551     (52) SRGFSDSGHLKYYVSPA----RCSGKK----------------------EKSKKK--QLKLLRRLSRDLPIFSYAVCGEEG-NG-SLIGEVKEKMISEAT
SlDGAT3-AK247265          (4) GCGFYDEGCLEYYSSGRGGIIRCGKKKNKE------------KDMETAELKKTKKKMETKLLKGLTKNLSNLNGMETGLGFGCDVDLVDQVQGKTISEAA
Consensus               (201)   GF D GHL YY                                        KK    K L   SKDL      G          L      K  SEA
```

```
                            301                                                                                                 400
      AhDGAT3-AY875644.1   (99) MVLMK--QLEQVRAEEKELKKKRKQEK-----------KEAKLKASKMNT---------NPDCESSSSSSSESESESSESECDNEVVDMKKNIKVGVAV
     GmDGAT3-XP_003542403   (95) EVLLR--ELEAARAKEKEMKKKRKQE-----------IKAKLKASKMN------------CESSSSSSSESS---DSDGDCDQVVDMNCFRAGAGVVV
        LjDGAT3-AFK37850  (110) EGLLK--ELEKLRAEEKELKKKRKDE-----------KKAKLKASKMKT---------GPDCESSSSSSSESSE--SSASECDEVVDMNTFRGGVAVAP
        MtDGAT3-ACJ86204  (104) EELLR--ELEKVRAEEKELKKKMKQEK-----------KKAKLKPSKMKT---------CNKSESSSSSSSESE---SSDSDCGEVVDMNTFRGAGVVDV
      AtDGAT3-AAD49767.1  (127) EILVK--QLEQLKAEEKILKKQRKEE------------KAKAKAMKKMT---------EMDSESSSSS-------ESSDSDCDKGKVVDMSSLRNKAKP
       AtDGAT3-NP_175264   (52) EILVK--QLEQLKAEEKILKKQRKEE------------KAKAKAMKKMT---------EMDSESSSSS-------ESSDSDCDKGKVVDMSSLRNKAKP
    AtDGAT3-XP_002891423  (127) EILVK--QLEQLKAEEKLLKKQRKEE------------KAKAKAMKKMT---------EMDSESSSSS-------ESSDSDRDKGKVVDMSSLRNKTKP
         VvDGAT3-CBI26023  (135) EVLLK--QLQQMRAEEKELKRRRKEE------------KAKLKATRMET---------GVVCESSSS--------ESSDSECGE--VVDMTHLRSGAVV
    VvDGAT3-XP_002269582  (135) EVLLK--QLQQMRAEEKELKRRRKEE------------KAKLKATRMET---------GVVCESSSS--------ESSDSECGE--VVDMTHLRSGAVV
    PtDGAT3-XP_002314335    (1) ---MK--QLGQLRAEEKELKRKKKEE------------KAKLKAVKMKT---------MLDCESSSSS-------ESSDSECGE--VIDMKRLRNEAVA
   RcDGAT3-XP_002519339.1 (107) QGLMK--QLQELRAKEKELKRQKKQE----------------KKAKLKS---------ESSS-SSSSE-------SSSDSERGE--VIHMSRFRDETIP
                  VfDGAT3   (97) QELIK--ELQELRAKEKELKRNKKQE------------KAKLKAEARIN---------YQCQSSTS--------ECSDSEC------DS-NLRNGVVQ
   BdDGAT3-XP_003568982.1  (93) ELLLL--ELNQMKDAEDDLKKREKEE------------KAAMKALKKQQ-KEAKKAAAVMTCGDGSS--------ESSESECEEEQSTEMSCVATKSMP
        HvDGAT3-BAJ97917   (94) ELLLQ--ELNQMKDAEDDLKKKQKEE------------KAAMKALKKQQ-KEAKKAT--MNCGDGSS--------ESSESECEEEQSMKMSCVATTSMP
        OsDGAT3-EAY96477   (90) ELLLE--ELNRMRDEEGELKKMKED------------KDAMKALKKQQ-KEAMKAATTMKCDDDDSSES--S--ESSESEC-EEQMMTMSCVASVTMP
     OsDGAT3-NP_001054585   (90) ELLLE--ELNRMRDEEGELKKMKED------------KDAMKALKKQQ-KEAMKAATTMKCNDDDSSES--S--ESSESEC-EEQMMTMSCVATVTMP
    SbDGAT3-XP_002439241   (91) DLLLQ--ELSQMKDEEYKIKKVEKEE------------KAAMKALKKQE-DATKTAIAMMKCEEDESSS------ESSESDCEDEATMNVDQRTMISTA
         ZmDGAT3-ACR36974    (1) ----------MKDAEYKINKVEKEQ------------NAAMKALKKQEKRTKKTAIMMMKCEEDESS-------ESSESDCEDEATMNVEQGLVISTA
     ZmDGAT3-NP_001183501   (89) DLLLQ--ELSQMKDAEYKINKVEKEQ------------NAAMKALKKQEKRTKKTAIMMMKCEEDESS-------ESSESDCEDEATMNVEQGLVISTA
     PpDGAT3-XP_001764081  (286) DLQVEVELLNAKKKKKKKKSRSSSSSSSDSSDYALSEAVAKLKLGNAKE--KRNK--KELSLSSSSSSSSSSSSSSSESNDMEIGKAVDLRALRKNKKC
     SmDGAT3-XP_002964239   (31) ELQLK------AKAKKKELKCKKEKEK-----------KQSAMAAGPAMK---------KRKKESSSSSSES---SDSSGGECGS-SRFDMSLLREELRA
         PgDGAT3-BT109069  (142) NVLMETRQLEQAKAERKERKQQLKAQK-----------KALKLAEKQRKN---------EGRCPESSSSSC----SSNHESYVVEMETALLHPTQQIGRI
         PsDGAT3-ABR16961  (132) NVLMR--QLEQAKAERKERKQQLKAQK-----------KALKLAEKQRKN---------EGRCPESSSSSC----SSNHESYVVEMA--LLHPTQQIGRI
         PgDGAT3-BT111835  (123) NVLMETRQFEQAKAERRERKRQLKAQK-----------KALKLAEKQRKN---------KGCCEDSSSS------SSDSESEAVDMETTLLRSTQQVGRI
         PsDGAT3-ABK23206  (117) NVLMG--QFEQAKTERKERKRQLKAQK-----------KALKLAEKQRKN---------KGCCEDSSSS------SSDSESEAVDMT--LLRSTQQVGRI
     PtDGAT3-XP_002301551  (122) EILLA--ELQNRRLERKEQKRKRRDES-----------ATLIKNRPRCDS---------GSSSSSSSSSGSSSPESSDSDSCSR-EVVSMKQMRSKALN
         SlDGAT3-AK247265   (92) ELLLG--QLQQLKAEEKELKRKRKEEK-----------AQMETKMETKVAASEVQSNTRSCAMETSSSSSSSSESSDSSDDECQNLVDMETKSLKIGTLA
                Consensus  (301) E LL    L Q KAEEKELK   KEE             KAK KA K                CESSSSS       ESS SEC

                            401                                                                                                 500
      AhDGAT3-AY875644.1  (177) AVADSPRKAETMILYTSLVARDVSANHHHHNAV-------------------------------ELFSRNNDISVGSINGGLKNE-------------
     GmDGAT3-XP_003542403  (165) PAPVEES-------PLPKTPIVEDTNAKAHRDA-------------------------------MALCSKNDISVSSVRDCIKSE-------------
        LjDGAT3-AFK37850  (185) APPPALP-------PSGPAALLPET-----------------------------------------FVGGDVSIGSVSVGLKNE-------------
        MtDGAT3-ACJ86204  (179) ATKPVDE-------LELKLKRPMLSIPEDSTSH-------------------------------HHVMDVCTTNNASLVTGFKKE-------------
      AtDGAT3-AAD49767.1  (196) VLEPLQPEATVATLPRIQEDAISCKNTSEALQI---------------------------------ALQTSTIFPSMANPGQTLK-------------
       AtDGAT3-NP_175264  (121) VLEPLQPEATVATLPRIQEDAISCKNTSEALQI---------------------------------ALQTSTIFPSMANPGQTLK-------------
    AtDGAT3-XP_002891423  (196) VLEPLQPEATVATLPRIQEDAQSCKNTSEALQI---------------------------------ALQTSTVFPSMPNPVQTLK-------------
         VvDGAT3-CBI26023  (201) EPIKDESQPVIQEAKGLEEP----------------------------------------------------------------------------
    VvDGAT3-XP_002269582  (201) EPIKDESQPVIQEAKGLEEPSLLQPVTTTLKGE---------------------------------CCTAVNTATSVA-----VD-------------
    PtDGAT3-XP_002314335   (65) EPIIGELQSVAQEEPTSILPALLTQESNVTEINGYHDHGLG---------------------IHGEECGGARSTSCSNAIRVSCN-------------
   RcDGAT3-XP_002519339.1 (169) AALP-QLHPLTHHHPTSTLPVSPTQECNPMDYT--STH--------------------------HEKRCCVGPSTGADNAVGDCCN-------------
                  VfDGAT3  (157) PVLVELQQQLEAIGHDTSSN--------------------------------------------------------------------------------
   BdDGAT3-XP_003568982.1 (169) GIAQGMVLPMSVPQITASEIGTIPAIEFDKAAMKAMRKMEKEQKKAAKKAMKMKKEEE-KRMAKLNSCKGDDSSSCSSESSDSECEGEVVRMSRCATITA
        HvDGAT3-BAJ97917  (168) AIEQGMVMSMSVPQITASNIARAPAMDFDKAAMKAMKKKEKEQKKAAKKAMKMKKEEE-KRMATLNSCK-DEDSSCSSESSNSECEGEVIRMSRCATISA
        OsDGAT3-EAY96477  (169) QIGEGVAISTTVPQVAASDVATAPAMEYDQAAMKAMKKREKEEKKAAKMAMKMKKKE--KKMATLTLCKDEDSTSCSSESSDSECE-EVVRMSRCATITT
     OsDGAT3-NP_001054585  (169) QIGEGVAISTTVPQVAASDVATAPAMEYDKAAMKAMKKREKEEKKAAKMAMKMKKKE--KKMATLTLCKDEDSTSCSSESSDSECE-EVVRMSCCATITT
    SbDGAT3-XP_002439241  (169) DPEVVASSILAVSAMECDKAAMKAMKKMEKEQMKAMKKMEK----AAKKAMKMEKEAKKMAMATLNGCRDEDDSSCSSESSESECEGQLVRMSRCVTITA
         ZmDGAT3-ACR36974   (70) VPEVVASSISAVPAMECDKAAVKAMKKMEKEQMKAMKKMEKEQKKAAKKAMKMEKEAKKMAMATLNGCRDEDDSSCSSESSESECEGQVMRMSRCVTITA
     ZmDGAT3-NP_001183501  (167) VPEVVASSISAVPAMECDKAAVKAMKKMEKEQMKAMKKMEKEQKKAAKKAMKMEKEAKKMAMATLNGCRDEDDSSCSSESSESECEGQVMRMSRCVTITA
     PpDGAT3-XP_001764081  (382) AKQFGFRFAINAPSNASVSAIDTVSLVPVESTSQLNQVVETMRSIPSIASAERTAVGIANLGSLSGNVHEQEPAIISQLRALCSTPSTCTSTN-------
     SmDGAT3-XP_002964239  (101) AASPRSIIDAVASEASLEQVN-----G-GK----------------------------------------------------------------------
         PgDGAT3-BT109069  (218) LEAPEPSPLVLIESPPNVAEVNGDEKALHVEFAEAS---------------------------------DRLGLNSGVD------------------
         PsDGAT3-ABR16961  (204) LEAPEPSPLVLIESPPNVAEVNGDEKALHVEFAEAS---------------------------------DRLGLNSGVD------------------
         PgDGAT3-BT111835  (197) LEAPEQSPSVLIESPPYVAEVKGDYKALHVEFAEAN---------------------------------DRLGLNSGMET---G-------------
         PsDGAT3-ABK23206  (187) LEAPEQSPSVLIESPPYVAEVKGDYKALHVEFAEAN---------------------------------DRLGLNSGMG------------------
     PtDGAT3-XP_002301551  (198) PFIEIESAKAIKEATQEDQHRDTVSGAKSNDSS--------------------------------PQNLSDG------------------------
         SlDGAT3-AK247265  (179) QTIPEACDRALDNATLNPSLSTPEVDTTVEISSMETPSTTTEESTGKTTSFEFSVPEQ-----KGECCLEASDCHIGNVGSSITPG-------------
                Consensus  (401)                                                                       S

                            501                                                                                                 600
      AhDGAT3-AY875644.1  (231) --------------------------N-----------------TAVITTEAIPQKRIEVCMG--NKCKKSGS--IALLQEFERVVGAEGG-AAAAVVGC
     GmDGAT3-XP_003542403  (212) ---------------------------------------------SAVVTAAPQKRIEVCMG--TKCKRSGA--AALMQEFERVVGVEG----GAVVSC
        LjDGAT3-AFK37850  (221) ---------------------------------------------NHGVSTAPQKRIEVCMG--NKCKKSGA--AALMQQFESVVGVEG----GAVVGC
        MtDGAT3-ACJ86204  (226) ---------------------------------------------TNVVIPTAQKRIEVCMG--NKCKKSGA--AALLQEFEKVVGVEGE---GVVVGC
      AtDGAT3-AAD49767.1  (248) --------------------------T-----------------VEAVSVVGLPLNRVEVCMG--GKCKRSGG--ALLLDEFQRAMTGFEG----SAVAC
       AtDGAT3-NP_175264  (173) --------------------------T-----------------VEAVSVVGLPLNRVEVCMG--GKCKRSGG--ALLLDEFQRAMTGFEG----SAVAC
    AtDGAT3-XP_002891423  (248) --------------------------T-----------------VEAVSVVGLPLKRVEVCMG--GKCKKSGG--ALLLDEFQRAMTGFQG----SAVAC
         VvDGAT3-CBI26023  (221) -----------------------------------------------MMGAGAKRIEVCMG--GKCKKSGA--EALLEEFERVVG-VEG----AVVGC
    VvDGAT3-XP_002269582  (248) --------------------------Q-----------------NEKTQVMGAGAKRIEVCMG--GKCKKSGA--EALLEEFERVVG-VEG----AVVGC
    PtDGAT3-XP_002314335  (129) --------------------------P-----------------TSSSMMSGTSDKRIEVCMG--NKCKKSGG--VALLEEFEKAVG-IGG----AVVGC
   RcDGAT3-XP_002519339.1 (226) --------------------------D-----------------RNSSMTEELSANRIEVCMG--NKCKKSGG--AALLEEFQRVLG-VEA----AVVGC
                  VfDGAT3  (177) ----------------------------------------------------LSKAQAKRIEVCMG--NKCKKSGG--AALLEAFESVVG-VEG----AVVGC
   BdDGAT3-XP_003568982.1 (268) PRTPPATTVFPIIVPQIPDSAALG-----AQISSGPTTAIQCTATSIAVVEKPALNRIEVCMG--GKCKKSGS--LTVLQEFEKQVGTGG-----AVVGC
        HvDGAT3-BAJ97917  (266) PRTPPASTVFPIIVPQIPDSVALE-----AQIFSGSANATECTATSIAVVEKPVLNRIEVCMG--GKCKKAGS--LAVLQEFETELGMGG-----TVVGC
        OsDGAT3-EAY96477  (266) PQTQPSSTVFPIILPQIPESVTLEPCQ-DAQISSEPANTMLSTTATIAVVEKPMTNRIEVCMG--GKCKKSGS--LAVLQEFEKKVGTDG-----AVVGC
     OsDGAT3-NP_001054585  (266) PQTQPSSTVFPIILPQIPESVTLEPCQ-DAQISSEPANTMLSTTATIAVVEKPMTNRIEVCMG--GKCKKSGS--LAVLQEFEKKVGTDG-----AVVGC
    SbDGAT3-XP_002439241  (265) PEMPSPTTGLPIIVPQIPAPLAPEPSQ----PLEPVTAVQATRVSSVAVAETSTTSRIEVCMG--GKCKKAGA--ALLQEFEKTVGTEG-----AVVGC
         ZmDGAT3-ACR36974  (170) PEMPPPSTGLPIIVPQIPEPLAPQPSQS--QPSEPATAVQATRVSSVAVTETSTTSRIEVCMG--GKCKKSGA--VALLQEFEKTVGTGG-----AVVGC
     ZmDGAT3-NP_001183501  (267) PEMPPPSTGLPIIVPQIPEPLAPQPSQS--QPSEPATAVQATRVSSVAVTETSTTSRIEVCMG--GKCKKSGA--VALLQEFEKTVGTGG-----AVVGC
     PpDGAT3-XP_001764081  (475) ----PS--------PIHSRGPIELKDTPGGRVIVETELSFLPSVVDPPPTTANTGGKIEVCTVG--KCRKGGS--QQILASLKSIVPESSN---ISVTSC
     SmDGAT3-XP_002964239  (125) ----------------------------------------------CVLAPPVCARVEVCTG--GKCRKAGS--QQVLAAFQAKISSSSSPSLSSATSC
         PgDGAT3-BT109069  (264) ----------------------------------------------IPLQEANSVIKVCMETGGKCKKSGSEMETLLEALEERISKLGLGCEVEAVGC
         PsDGAT3-ABR16961  (250) ----------------------------------------------IPLQEANSVIKVCMG--GKCKKSGS--EMLLEALEERISKLGLGCEVEAVGC
         PgDGAT3-BT111835  (245) ----------------------------------------------IPVQEVNSVIKVCMETSGKCKKSGSEMETLLGAFEERINKSGIGFDVEAVGC
         PsDGAT3-ABK23206  (233) ----------------------------------------------IPVQEVNSVIKVCMS--GKCKKSGS--EMLLGAFEERINKSGIGFDVEAVGC
     PtDGAT3-XP_002301551  (238) ----------------------------------------------VQIGASGRKIEICMG--GKCRKLGA--AALLEEFERKIGMES-----AVVGC
         SlDGAT3-AK247265  (260) ------------------------------------TRSNVSSIAAATTTTAEGTKRIEVCMETGGKCKRLGAG--ALLEEFQRVAGIEA-----AVSGC
                Consensus  (501)                                                     RIEVCMG  GKCKKSG    ALL EFE   VG  G      AVVGC
```

```
                              601                                                                                              700
    AhDGAT3-AY875644.1  (283) KCMG--KCKSAPNVRIQNSTAD---K---IAEGFNDSVKVPA-NPLCIGVAWRMLKPLWLRFLGENQESTNE----------------------------
    GmDGAT3-XP_003542403  (258) KCMG--KCKTAPNVKVQNSVDH---S---LARGLDDSVNIPA-NPLCIGVGLGDVDAIVARFLGESHTDIGMIGAATAT---------------------
      LjDGAT3-AFK37850  (267) KCMG--KCKSAPNVRIQNAVDH---E---LAEGLNDSVKVPA-NPLCIGVGLEDVDAVVARFLGENYQDMGMVAATT-----------------------
      MtDGAT3-ACJ86204  (273) KCMG--KCKTAPNVRIQNSVDL---N---MVQGIDDSVKIPS-NPLCIGVGLEDVDTIVARFLGEDYKDVGMVAAASS----------------------
    AtDGAT3-AAD49767.1  (297) KCMG--KCRDGPNVRVVKETDA----------VMTDSVRTPS-KTLCVGVGLQDVETIVTSFFDEECSREGLGSVSY-----------------------
      AtDGAT3-NP_175264  (222) KCMG--KCRDGPNVRVVKETDA----------VMTDSVRTPS-KTLCVGVGLQDVETIVTSFFDEECSREGLGSVSY-----------------------
  AtDGAT3-XP_002891423  (297) KCMG--KCRDGPNVRVVNETDS----------VMTDSVRTPS-KTVCVGVGLQDVETIVTSFFDEECSREGLGYVSH-----------------------
      VvDGAT3-CBI26023  (263) KCMG--KCRVGPNVRVLNSIEG---KPMKILDCLHQLRRPFF-FFIFCGYTKSNGRYVPCLFFFYLFYFYFLGFGLLWRMLKGNNPFVNVNIRFGQLPYS
  VvDGAT3-XP_002269582  (296) KCMG--KCRVGPNVRVLNSIEG------VEAEGMDDSVRTPA-NPLCVGVGLQDVGIIVSNFFGETHEDIGLPAPA------------------------
  PtDGAT3-XP_002314335  (177) KCMG--KCRDGPNVRILKSGNE----------GVDDSVRIPAANPLCIGVGLEDVDVIVANFFGKELSVALAT---------------------------
RcDGAT3-XP_002519339.1  (274) KCMG--NCRDGPNVRVRNSVQD---R------NTDDSVRTPS-NPLCIGVGLEDVDVIVANFFGLGLAPAS-----------------------------
              VfDGAT3  (219) KCMG--KCRDGPNVRVNAAHDP-------------ITTPPNS-YPLCIGVGLEDVGVIVANYFQDKDKHLGLAPAS------------------------
BdDGAT3-XP_003568982.1  (354) KCLG--KCGVGPNVRLRSEVPE---E-----------GSLQNKSTLCIGVGLEDVGTIVAGFFGDDSDLLGMN---------------------------
      HvDGAT3-BAJ97917  (352) KCLG--KCGLGPNVRLRSEG------------------SVQKNSPICIGVGLGDVGTIVAGFLGDG--DLGMN---------------------------
      OsDGAT3-EAY96477  (356) KCLG--KCGLGPNVRLRSEG------------------AAQK-NPLFIGVVLEDVGTIASGLFGGG-DDLGMVNNLAQPLPYL-----------------
  OsDGAT3-NP_001054585  (356) KCLG--KCGLGPNVRLRSEG------------------AAQKKNPLFIGVVLEDVGTIASGLFGGG-DDLGMVNN-------------------------
  SbDGAT3-XP_002439241  (352) KCLG--KCGLGPNVRLRSDISA---E-----------GSAKG-SPLCIGVGFEDVSTIVAGLFGDA--DLGMTPT-------------------------
      ZmDGAT3-ACR36974  (259) KCLG--KCGLGPNVRLRSGASA---E-----------GSAKG-NPLCIGVGFEDVGTIVAGLFGGA--DLGMTPT-------------------------
  ZmDGAT3-NP_001183501  (356) KCLG--KCGLGPNVRLRSGASA---E-----------GSAKG-NPLCIGVGFEDVGTIVAGLFGGA--DLGMTPT-------------------------
  PpDGAT3-XP_001764081  (556) KCMG--KCKSAPNVRVKNSERQ---------------------SHLHSHVSEDDADTLFDYHFGLQPSGNRLPALVASINSDFSAAV-------------
  SmDGAT3-XP_002964239  (174) KCMG--MCGQGPNVRIQGAGDG--------------------APLAFNHVGVEDVDPLLQQAGFPAVVAVTRMQSARY----------------------
      PgDGAT3-BT109069  (316) KCMETGKCRNAPNVRVQTEEDG--------------FHGGKGEVHMETGVDIGDVDLILAQHFGLNLQLPQQPK--------------------------
      PsDGAT3-ABR16961  (298) KCMG--KCRNAPNVRVQTEEDG--------------FHGGKGEVHMG--VDIGDVDLILAQHFGLNLQLPQQPK--------------------------
      PgDGAT3-BT111835  (297) KCMETGKCRNAPSVRVQTEED---------------VGKGVMETHMETGVNIGDIDLILAQHFGLNLQQPPSPKEGQTAT--------------------
      PsDGAT3-ABK23206  (281) KCMG--KCRNAPSVRVQTEED---------------VGKGVMH----MGVNIGDIDLILAQHFGLNLQQPPSPKEGQTAT--------------------
  PtDGAT3-XP_002301551  (281) KCMG--KCMKGPNVRVFNCTVE---N----EDMRVEDSIKPPLNLLCIGVGLKDVGIISRQLLGNDGKDRNHACLMAPS---------------------
      SlDGAT3-AK247265  (317) KCMETGKCKVGPNVKVSGCSSSSISDAFQAGDSVAVSSAPTTSNSLCIGVGLEDVSLIAANLLGRYQEVGLANALP------------------------
             Consensus  (601) KCMG  KC  GPNVRV                            PLCIGVGL DV  IVA  FG      G


                              701        714
    AhDGAT3-AY875644.1  (346) --------------
  GmDGAT3-XP_003542403  (328) --------------
      LjDGAT3-AFK37850  (335) --------------
      MtDGAT3-ACJ86204  (342) --------------
    AtDGAT3-AAD49767.1  (361) --------------
      AtDGAT3-NP_175264  (286) --------------
  AtDGAT3-XP_002891423  (361) --------------
      VvDGAT3-CBI26023  (357) EDILLFTKMILSSN
  VvDGAT3-XP_002269582  (363) --------------
  PtDGAT3-XP_002314335  (238) --------------
RcDGAT3-XP_002519339.1  (333) --------------
              VfDGAT3  (279) --------------
BdDGAT3-XP_003568982.1  (411) --------------
      HvDGAT3-BAJ97917  (403) --------------
      OsDGAT3-EAY96477  (417) --------------
  OsDGAT3-NP_001054585  (410) --------------
  SbDGAT3-XP_002439241  (408) --------------
      ZmDGAT3-ACR36974  (315) --------------
  ZmDGAT3-NP_001183501  (412) --------------
  PpDGAT3-XP_001764081  (620) --------------
  SmDGAT3-XP_002964239  (230) --------------
      PgDGAT3-BT109069  (376) --------------
      PsDGAT3-ABR16961  (354) --------------
      PgDGAT3-BT111835  (362) --------------
      PsDGAT3-ABK23206  (340) --------------
  PtDGAT3-XP_002301551  (351) --------------
      SlDGAT3-AK247265  (393) --------------
             Consensus  (701)
```

Figure S2. Identification of amino acid residues and sequence motifs conserved in DGAT3s. Each DGAT sequence name is on the left of the alignment followed by the position of amino acid residue of DGAT protein sequence in the alignment. The letters at the bottom of the alignment are the consensus residues. Color codes for amino acid residues are as follows: 1) red on yellow: consensus residue derived from a completely conserved residue at a given position; 2) blue on cyan: consensus residue derived from the occurrence of greater than 50% of a single residue at a given position; 3) black on white: non-similar residues. The abbreviations of the organisms are: Ah, *Arachis hypogaea* (peanut); At, *Arabidopsis thaliana*; Bd, *Brachypodium distachyon;* Gm, *Glycine max* (soybean); Hv, *Hordeum vulgare (barley)*; Lj, *Lotus japonicas*; Mt, *Medicago truncatula*; Os, *Oryza sativa* (rice); Pg, *Picea glauca* (white spruce); Pp, *Physcomitrella patens*; Ps, *Picea sitchensis* (sitka spruce); Pt, *Populus trichocarpa*; Rc, *Ricinus communis* (caster bean); Sb, *Sorghum bicolor* (sorghum); Sl, *Solanum lycopersicum* (tomato); Sm, *Selaginella moellendorffii*; Vf, *Vernicia fordii* (tung tree); Vv, *Vitis vinifera* (grape); Zm, *Zea mays* (corn).
